# Arjunolic Acid From *Terminalia ivorensis* A. Chev (Combretaceae) Possesses Anti‐Breast Cancer Effects In Vitro and In Vivo

**DOI:** 10.1002/cnr2.70337

**Published:** 2025-09-04

**Authors:** Muriel Angounou Akamse, Vigny Diembo Kamgo, Patrick Yves Ango, Marthe Carine Djuidje Fotsing, Boris Hugor Pehuie Fomat, Edwin Mmutlane, Ghislain Wabo Fotso, Deccaux Wabo Fotso Gilbert Kapche, Stephane Zingué, Derek Tantoh Ndinteh

**Affiliations:** ^1^ Department of Organic Chemistry, Faculty of Science University of Yaoundé 1 Yaoundé Cameroon; ^2^ Department of Pharmacotoxicology and Pharmacokinetics, Faculty of Medicine and Biomedical Sciences University of Yaoundé 1 Yaoundé Cameroon; ^3^ Centre for Natural Product Research (CNPR), Department of Chemical Sciences University of Johannesburg Johannesburg South Africa; ^4^ Research Centre for Synthesis and Catalysis, Department of Chemical Sciences University of Johannesburg‐Kingsway Campus Auckland Park South Africa; ^5^ Department of Chemistry Higher Teacher Training College, University of Yaoundé 1 Yaoundé Cameroon

**Keywords:** anti‐inflammatory, arjunolic acid, breast cancer, cytokines, DMBA, *Terminalia ivorensis*

## Abstract

**Background:**

Breast cancer is a major public health issue. In 2022, approximately 4,207 new cases and 2,285 deaths were reported in Cameroon. Given the limited accessibility and various issues associated with conventional treatments, herbal medicine has emerged as a promising alternative. Aims: This study aimed to evaluate the potential anticancer activity of naturally occurring compounds isolated from Terminalia ivorensis A. Chev.

**Methods and Results:**

This was done by fractionating the methanolic extract of T. ivorensis and purifying the constituents obtained using conventional chromatographic techniques. Thereafter, the crude extract and its 5 isolates were subjected to in vitro MTT bioassay to assess their potential to kill human (MCF‐7 and MDA‐MB‐231) and murine (4T1) breast cancer cell lines. Furthermore, the potential of the most active compound (arjunolic acid) to mitigate DMBA‐induced breast cancer in rats was tested. Treatments were administered for a period of 121 days; the group of rats treated with arjunolic acid (1 mg/kg) was compared to the group that received tamoxifen at 3.3 mg/kg (standard), as well as to the normal and negative control groups. Key parameters assessed included survival, tumor burden, cytokine profiles, as well as hematological, hepatic, and renal functions. Out of the 5 isolates [lupeol (1), betulinic acid (2), Arjunolic acid (3), 3,3'‐Di‐O‐methylellagic acid‐4'‐O‐β‐D‐glucopyranoside (4) 3,3’,4’‐Tri‐O‐methylellagic acid‐4‐O‐β‐Dglucopyranoside (5)] from T. ivorensis, compound (3) had the most significant inhibitory effect against breast cancer cells growth with an average CC50 of 20 μg/mL. In vivo, a significant reduction (~89%) in tumor burden and favorable modulation of inflammation, characterized by a decrease in pro‐inflammatory cytokines (TNF‐α, IFN‐γ, IL‐6, VEGF) and an increase in anti‐inflammatory IL‐10 was observed. Moreover, treatment with arjunolic acid led to improved survival and maintenance of body weight, without inducing any notable adverse effects.

**Conclusion:**

Arjunolic acid should receive more attention as a candidate for an effective therapeutic option, combining anticancer effects with beneficial anti‐inflammatory activity. We encourage further studies on this compound to better understand its mode and mechanism of action.

**Abbreviations**
AA: arjunolic acid
ALP: alkaline phosphatase
ALT: alanine aminotransferase
ANOVA: analysis of variance
AST: aspartate amino transferase
ATC: American type culture collection
BC: breast cancer
BW: body weight
COSY: correlation spectroscopy
DMBA: 7,12‐dimethylbenz(a)anthracene
ELISA: enzyme‐linked immunosorbent assay
HMBC: heteronuclear multiple bond correlation
MeOH: methanol
mp: melting point
MQC: heteronuclear multiple quantum coherence
MTT: mitochondrial tetrazolium test
NMR: nuclear magnetic resonance
PAHs: polycyclic aromatic hydrocarbons
PLT: platelets
RBC: red blood cell count
rpm: revolution per minute
RPMI‐1640: Roswell park Memorial Institute
TLC: thin layer chromatography
WBC: white blood cell count

## 1 Introduction

Breast cancer (BC) continues to pose a significant global public health concern, ranking as the most frequently diagnosed malignancy among women. According to 2022 data from GLOBOCAN, approximately 2.3 million new cases were reported worldwide, accompanied by an estimated 685,000 deaths [1]. Representing 11.7% of all cancer cases globally, BC disproportionately affects populations in low‐ and middle‐income countries, where incidence and mortality are projected to escalate, potentially exceeding 3 million new diagnoses and 1 million deaths by 2040 if effective preventive strategies are not implemented [2]. These figures highlight the scale of a problem exacerbated by geographical and socioeconomic disparities that influence incidence and mortality rates across the world, with population growth and aging being the major causes [3]. In countries like Cameroon, the impact of this disease is intensified by limited resources and frequent late‐stage diagnoses [4]. The pathogenesis of cancer is not fully understood; however, the initiation of cancer is primarily attributed to alterations in cellular function and genetic mutations. These genetic disruptions are often driven by environmental stressors, tobacco exposure, and a range of carcinogenic substances, all of which contribute to the transformation of normal cells into malignant ones [5].

Currently available treatments include local approaches (surgery, radiotherapy, cryotherapy) and systemic therapies (chemotherapy, hormone therapy, immunotherapy, targeted therapy) that can be used individually or in combination. Despite significant advances, these therapies—systemic therapies in particular are—associated with adverse effects, as they are administered through the bloodstream, and can therefore exert various effects on the normal cells in body [6]. Moreover, prohibitive costs, mainly for those in low‐ and middle‐income countries like Cameroon, limit accessibility to these treatments for a large portion of the population. In this context, the search for alternative therapies, particularly those derived from herbal medicine, stands as a critical avenue for advancing cancer therapy in these countries [7]. Traditional medicine practiced in Africa emphasizes the use of medicinal plants which, despite a long history of empirical use, are now increasingly supported by rigorous scientific studies [8]. Among the resources explored is *Terminalia ivorensis* , which is the subject of this study.

*Terminalia ivorensis* A. Chev (Combretaceae) is a species native to West and Central Africa, which forms a good taproot supported by 6 to 8 powerful lateral roots but also a widespread and rather superficial root system. It can reach a height of 46 m, with no branches up to 30 m, and a diameter of between 2 and 4.75 m. Mature trees have a very flat top with a broad horizontal canopy of evenly distributed foliage, starting from the apex of the straight trunk. The bark is smooth and light grey to dark brown on young trees and branches, and often blackish with deep longitudinal cracks on mature trees [9]. This plant is reported to be useful in the treatment of cuts, ulcers, wounds, hemorrhoids, sores, diuresis, general body pains, malaria, and yellow fever [10, 11]. Numerous in vitro studies have also demonstrated *T. ivorensis* 's antifungal, antioxidant, antibacterial, and anti‐plasmodial properties [12, 13, 14]. Previous in vivo reports on *T. ivorensis* A. Chev. show that its ethanolic stem bark extract could protect against gentamicin‐induced renal and hepatic damage in rats [15]. Furthermore, this extract has also been shown to protect rats against potassium dichromate‐induced nephrotoxicity [16]. The *Terminalia arjuna* bark extract showed growth inhibition against liver HEP2 (78%) and colon HT29 (79.33) cancer cell lines and alleviated hepatocellular carcinoma burden in rats [17, 18]. Various compounds have been identified in *T. ivorensis* , including acetophenone, benzoic acid, ellagic acid, 3′‐Di‐O‐methylellagic acid, cinnamic acid, flavonoids, indole, lignans, and triterpenes such as arjunic acid, oleanolic acid, arjugenin, sericoside, seric acid, ivorengenin A, ivorengenin B, ivorenoside A, ivorenoside B, ivorenoside C, betulinic acid, and punicalin [19, 20].

To the best of our knowledge, no study dealing with the anticancer potential of *T. ivorensis* has been reported yet. This justifies the present study, which aimed at evaluating the anticancer effect of *T. ivorensis* extract and isolates using MTT bioassays and a DMBA‐induced breast cancer rat model. The evaluated parameters included; in vitro cytotoxicity and in vivo survival and tumor burden reduction, cytokine profiles, hematological parameters, as well as hepatic and renal functions. The results could provide scientific evidence to enhance the potential of African biodiversity to treat BC and thus contribute to the integration of herbal medicine into modern breast cancer management strategies.

## 2 Materials and Methods

### 2.1 Plant Material

#### 2.1.1 Plant Collection

The stem bark of *T. ivorensis* was collected in Nkolbisson, Centre Region of Cameroon, in March 2022. The plant was taxonomically identified at the National Herbarium in Yaoundé, Cameroon, where a voucher specimen was deposited under the reference number 43982/HNC.

#### 2.1.2 Extraction and Purification

The air dried and powdered stem barks (700 g) were extracted by maceration with methanol (MeOH) for 48 h at room temperature. The extract was then concentrated under reduced pressure to give 170 g of a brown residue that constituted the crude extract. Part of this crude extract (150 g) was fractionated by using silica gel 60 (0.04–0.063 mm, 120 g) vacuum column chromatography using as eluent, hexane, hexane/CHCl_3_ 1:1 mixture, CHCl_3_ and CHCl_3_/MeOH. Fractions of 500 mL each were collected, concentrated under vacuum, and pooled on the basis of the thin layer chromatography (TLC) analysis to six fractions, A‐F. Compounds (**1**) lupeol [21] and (**2**) betulinic acid [22] were directly obtained from fractions B and C eluted with hexane/CHCl_3_ 7:3. The fraction D was subjected to column chromatography and eluted with isocratic DCM‐MeOH (95:5) to afford compound (**3**) arjunolic acid [23]. Fraction E was purified with chloroform‐methanol (95‐5) using silica gel column chromatography to afford two colourless amorphous solids (**4**) 3,3′‐Di‐O‐methylellagic acid‐4′‐O‐β‐D‐glucopyranoside and (**5**) 3,3′,4′‐Tri‐O‐methylellagic acid‐4‐O‐β‐D‐glucopyranoside [24]. Characterization of the isolated is as follows.

Compound 1 was obtained as a white solid in the Hex/AE system (1.5:8.5) with a melting point of 214°C–216°C. The presence of a hydroxyl functional group and an olefinic moiety was identified in the IR spectrum (Figure S1) at 3388.2 and 1658.7 cm^−1^, respectively. The carbon‐13 NMR spectrum (Figure S4) revealed 30 carbon signals. Signals from 0 to 55 ppm correspond to methine, methylene, and methyl carbons. The deshielded signal at 79.0 ppm is characteristic of an oxymethine carbon atom. The signals at 109.3, 25.1, and 150.9 ppm are typical of olefinic carbon atoms. Spectral analysis and comparison with reported data led to the conclusion that the structure of 1 is lupeol, a pentacyclic triterpene.

Compound 2 was obtained as a white solid with a melting point of 294 to 297°C. Its IR spectral data (Figure S6) showed a prominent peak at 1684 cm^−1^, indicating the presence of a C=O bond, as well as other bands appearing in the fingerprint region. The 1H NMR spectrum (Figure S8) showed peaks at 1.81 (2H), 1.53 (2H) and 2.52 (2H), as well as singlets at 2.68 and 1.98 (1H each). A pair of singlets at −5.44 and 5.56 ppm (1 H each) was due to the presence of vinyl protons at carbon position 29. Comparing our isolated compound with the sample present in the laboratory by TLC allowed us to confirm that compound 2 is betulinic acid.

Compound 3 was obtained as a white solid using the Hex/AE (3:7) solvent system with a melting point of 332°C–335°C. It is soluble in dimethyl sulfoxide (DMSO) and responds positively to the Libermann‐Burchard test, producing the characteristic purplish‐red colouration of triterpenes. Its (‐)‐HR‐ESI‐MS spectrum (Figure S10) shows two pseudo‐molecular ions, [M‐H]^−^ at m/z 487.3437 and [M + HCOO]^−^ at m/z 533.3513. This suggests the crude formula C_30_H_48_O_5_ for 3, containing seven degrees of unsaturation. Complete analysis of its NMR spectral data (^1^H NMR, Figure S11 ^13^C NMR, Figure S12; and 2D NMR, COSY Figure S13) allowed us to assign the structure of 3, which is arjunolic acid.

Compound 4, (C_22_H_20_O_13_) was isolated as a yellow oil. The ^1^H NMR spectrum (Figure S15) of Compound 4 showed a number of signals characteristic of sugars and ellagic acid. Two H singlets at *δ*_*H*_ 7.74 and 7.50 represented the aromatic H‐5′ and H‐5. The anomeric proton appeared as a doublet at *δ*_*H*_ 5.15 (J = 7.7 Hz). Two 3H singlets at *δ*_*H*_ 4.09 and 4.05 were most probably due to the two methoxy groups. The ^13^C NMR spectra (Figure S16) of compound 4 showed resonances for all twenty‐two carbon atoms, including two methyl, one methylene, seven methine, and twelve quaternary carbons. The chemical shifts of the 10 aromatic quaternary carbons in the 13C‐NMR spectrum led us to conclude that the compound is a highly substituted biphenyl derivative. The signals for the two lactone carbonyls (C‐6a and C‐6a′) resonated at *δ*_*C*_ 158.6 and 158.0, respectively, indicating that the compound has an ellagic acid skeleton. Similarly, the appearance of an anomeric carbon at *δ*_*C*_ 101.8, together with five oxygen‐bearing carbons resonating between *δ*_*C*_ 76.2 and 61.1, further confirmed the presence of a sugar moiety in the molecule. Based on the above spectral observations, compound 4 was deduced to be 3,3′‐Di‐O‐methylellagic acid‐4′‐O‐β‐D‐glucopyranoside.

Compound 5 (C_22_H_22_O_13_), was also isolated as a colorless solid with a melting point of 275°C–277°C. Its NMR (Figure S19) spectroscopic data were compared with those obtained from compound 4. The two sets of data were very similar. In the spectroscopic data of compound 5, three methyl group signals were found instead of the two found in compound 4; the rest of the data were similar. Therefore, compound 5 was identified as 3,3′,4′‐Tri‐O‐methylellagic acid‐4‐O‐β‐Dglucopyranoside.

#### 2.1.3 General Procedure

Thin‐layer chromatography (TLC) was performed using aluminum sheets pre‐coated with silica gel 60 F254 (Merck). Visualization of the spots was achieved under ultraviolet light at 254 and 366 nm, and by spraying with 50% sulfuric acid (H_2_SO_4_). Nuclear Magnetic Resonance (NMR) spectra were recorded on Bruker Avance 400 and 600 instruments, operating at 400 MHz and 600 MHz for ^1^H NMR, and at 75 and 150 MHz for ^13^C NMR, respectively. Residual solvent signals were used as internal references. Melting points were measured using a Kofler micro hot‐stage apparatus.

Mass spectrometric analyses were carried out with an API QSTAR Pulsar mass spectrometer. Structural elucidation of the isolated compounds was performed by comparing the obtained spectral data with those reported in the literature.

The melting points were determined on an electronic melting point apparatus. The melting point was taken as a range of the beginning to total melting temperatures.

### 2.2 In Vitro Study

#### 2.2.1 Cell Culture

Several breast adenocarcinoma cell lines were utilized in this study, including the murine mammary carcinoma cell line 4 T1, as well as the human estrogen receptor‐positive MCF‐7 and triple‐negative MDA‐MB‐231 cell lines, all obtained from the American Type Culture Collection (ATCC, Wesel, Germany). Reagents such as streptomycin, fetal bovine serum (FBS), GlutaMAX, penicillin, and HEPES were procured from Gibco/Invitrogen (Karlsruhe, Germany).

Cells were maintained in Roswell Park Memorial Institute (RPMI‐1640) medium supplemented with HEPES, 100 μg/mL streptomycin, 10% FBS, and 100 μg/mL penicillin. Cultures were incubated in a humidified atmosphere containing 5% CO₂ at 37°C and a pH of 7.4. For routine subculturing, 90% of the culture medium was refreshed with new medium every 2 days.

#### 2.2.2 Cytotoxicity Bioassay

Cell viability was assessed using the MTT assay (3‐(4,5‐dimethylthiazol‐2‐yl)‐2,5‐diphenyltetrazolium bromide). Breast cancer cells, both treated and untreated, were seeded into 96‐well tissue culture plates at a density of 1 × 10^4^ cells/mL in a volume of 100 μL per well. Test compounds were freshly prepared in 0.01% DMSO and evaluated at concentrations ranging from 2.5 to 100 μg/mL, whereas the crude extract was tested across a concentration range of 12.5 to 200 μg/mL. Control wells included untreated cells exposed to either 0.01% DMSO or RPMI‐1640 medium, while doxorubicin was used as the positive control. After 24 and 48 h of incubation, MTT solution (0.5 mg/mL) was added to each well, followed by an additional 4‐h incubation period. The resulting formazan crystals were solubilized using a lysis buffer composed of 10% SDS in 0.01 M HCl. The plates were then incubated overnight at 37°C in a humidified 5% CO₂ atmosphere. Absorbance was measured at 570 nm using a microplate ELISA reader to determine cell viability.

### 2.3 Assessment of the Chemopreventive Effect of Arjunolic Acid

#### 2.3.1 Animal Population and Housing Conditions

For this study, thirty‐two (32) healthy female Wistar rats, aged 4 to 6 weeks and weighing between 60 and 80 g, were obtained from the animal house of the laboratory of Animal Physiology (Faculty of Science, University of Yaoundé 1). Once in the animal house of the Basic and Clinical Cancer Research Unit (Faculty of Medicine and Biomedical Sciences, University of Yaoundé 1), a 10‐day acclimatization period was observed before the beginning of the experiment. The animals were housed at room temperature in groups of 4 in plastic cages with bedding (white wood shavings) and metal mesh tops. The rats had unlimited access to standard food and filtered water. Rat feed composition was: corn flour (42%), wheat flour (20%), fish meal (20%), groundnuts (peanuts) (9%), palm kernel cake (5.3%), bone meal (3%), premix (vitamin and mineral concentrate) (0.7%).

These conditions were carefully controlled to minimize stress and environmental variables that could influence the response to the treatments. The entire experimental protocol was developed in accordance with the European Union guide for the care and use of laboratory animals for scientific research (Directive 2010/63/EU). These protocols were endorsed by the Cameroon Institutional National Ethic Committee under the Ministry of Scientific Research and Technology Innovation (Registration No. FWA‐IRD 0001954).

#### 2.3.2 Breast Cancer Induction

Breast cancer was induced in this study using the environmental carcinogen DMBA (a polycyclic aromatic hydrocarbon‐PAH) at 50 mg/kg BW following 1 week of pretreatment with tested substances as described by Nguedia et al. [25]. In brief, DMBA was dissolved in 0.3 mL of olive oil and injected into the mammary glands air pouch of prepubertal rats aged ~60 days, while rats in the normal group received sham injections of olive oil only. After injecting rats with DMBA, they were treated with a solution of amoxicillin by oral route for 10 days to reduce pain and prevent infections.

#### 2.3.3 Experimental Group Distribution and Treatment Administration

The rats were randomly assigned to four groups (*n* = 8) as follows: normal control group (NOR) comprising rats not exposed to DMBA, receiving only the vehicle (distilled water). Negative control group, comprising rats exposed to DMBA and receiving the vehicle (distilled water). The third group was the positive control group (TAMOX) comprising rats exposed to DMBA and treated with tamoxifen (hormone therapy prescribed to women as a secondary prevention measure against breast cancer) at 3.3 mg/kg, and the last group was made up of rats exposed to DMBA and treated with arjunolic acid at a dose of 1 mg/kg. After verification of the compound's solubility and stability in the chosen vehicle, the drug preparations were made under sterile conditions. All treatments were administered daily by oral route for 121 days, with regular monitoring to ensure dose consistency and adherence to the protocol.

#### 2.3.4 Sample Collection, Processing, and Storage

At the conclusion of the treatment period, animals were humanely euthanized using an anesthetic combination of ketamine (50 mg/kg body weight, intraperitoneally) and diazepam (10 mg/kg body weight, intraperitoneally), in compliance with established ethical guidelines for animal sacrifice. Blood samples were collected at the time of euthanasia into both dry and EDTA‐coated tubes for subsequent biochemical and hematological analyses, respectively, as previously described [25]. Selected organs—including the liver, kidneys, spleen, lungs, heart, adrenal glands, uterus, ovaries, femur—and tumor tissues were promptly excised and fixed in 10% formalin for histopathological examination.

#### 2.3.5 Clinical Follow‐Up and Evaluation Criteria

A rigorous monitoring system was implemented to evaluate the parameters of interest:

- *Survival and body weight follow‐up*: animal weights were recorded on a weekly basis using a lab balance. Survival was recorded daily to establish a Kaplan‐Meier survival curve and identify any potential toxic effects.
- *Tumor evaluation*: rats were palpated twice a week to identify any signs of mammary tumor development. Tumors were measured on the rat using a digital caliper to determine their diameter and length, and tumor volume was calculated according to the formula (length × width^2^)/2 [26]. Tumor inhibition index was then determined for each group.
- *Histopathological analyses*: at the end of the study, the excised tumors were fixed in 10% formalin for 72 h, then dehydrated, embedded in paraffin, and sectioned using a microtome (thickness 5 μm). The sections were stained with hematoxylin and eosin (H&E) to confirm the cancerous nature of the lesions and evaluate the morphological changes induced by the treatment. The alterations in histological architecture observed in tumors and organs were examined by a pathologist in our Institution, and photomicrographs were captured using a light Axioskop 40 microscope equipped with a digital Celestro‐44421 camera. These images were transferred to a computer for analysis using Image J software.
- *Cytokine bioassays*: arteriovenous blood samples collected in dry tubes were centrifuged (3000 rpm for 15 min at 4°C), and serum separated. The concentrations of key cytokines (TNF‐α, INF‐γ, IL‐6, VEGF, and IL‐10) were quantified by Enzyme‐Linked ImmunoSorbent Assays (ELISA) using commercially validated kits, strictly following the manufacturer's protocols (including detection ranges and internal controls) as previously reported [25].
- *Hematological and biochemical parameters*: a complete blood analysis was performed on the blood collected in EDTA tubes using an automated hematological analyzer. The parameters evaluated included red blood cell count (RBC), white blood cell count (WBC), hemoglobin, hematocrit, and platelets. Concurrently, biochemical assays (AST, ALT, ALP, bilirubin, creatinine, urea) were conducted to assess liver and kidney functions using colorimetric kits (bought from Bio‐diagnostic Co. (Giza, Egypt)) following the manufacturer's instructions.

#### 2.3.6 Statistical Analysis

Data analysis was performed using GraphPad Prism version 8. The normality of data distribution was assessed using the Shapiro–Wilk test. Group comparisons were conducted using one‐way Analysis of Variance (ANOVA), followed by Dunnett's post hoc test for multiple comparisons. A *p*‐value of less than 0.05 was considered indicative of statistical significance. Results are expressed as mean ± standard error of the mean (SEM).

## 3 Results

### 3.1 Isolates From *Terminalia ivorensis* Methanolic Stem Barks Extract

**FIGURE 1:**
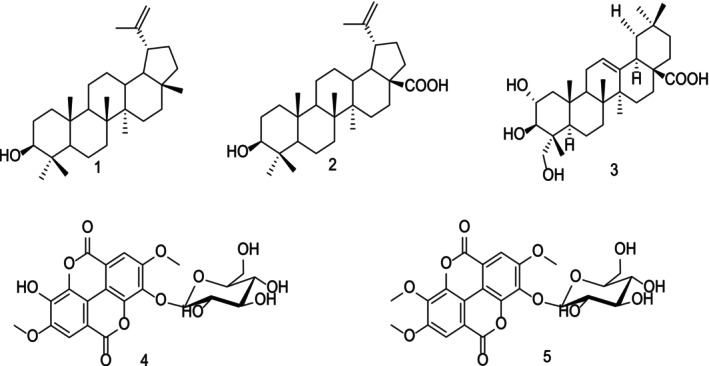
Structures of isolates from *Terminalia ivorensis* A. Chev.

The chemical structures of the isolated compounds are presented in Figure 1. Structural elucidation was achieved primarily through spectroscopic techniques, notably Nuclear Magnetic Resonance (NMR) spectroscopy, including two‐dimensional experiments such as COSY (Correlation Spectroscopy), HMQC (Heteronuclear Multiple Quantum Coherence), and HMBC (Heteronuclear Multiple Bond Correlation). These data (Supporting Information figures) were interpreted in conjunction with published literature and, in certain cases, compared with authentic reference compounds previously isolated and characterized within our research group.

The compounds isolated from the stem bark of *T. ivorensis* (Figure 1) were identified as lupeol (**1**), betulinic acid (**2**), arjunolic acid (**3**), 3,3′‐Di‐O‐methylellagic acid‐4′‐O‐β‐D‐glucopyranoside (**4**), and 3,3′,4′‐Tri‐O‐methylellagic acid‐4‐O‐β‐Dglucopyranoside (5). The triterpenoids (compounds **1**–**3**) are commonly found across a wide range of plant species, whereas ellagic acid derivatives such as compounds **4** and **5** have previously been reported in other species of the Terminalia genus.

### 3.2 Cytotoxicity of *Terminalia ivorensis* Crude Extract and Its Isolates on Breast Cancer Cells

Table 1 shows the cytotoxic potential of the *T. ivorensis* methanolic extract as well as that of its isolated compounds. Of the three cell lines tested, 4 T1 murine cells appear more sensitive than human cells. *T. ivorensis* methanolic extract exhibited weak cytotoxicity on the three cell lines with CC_50_ values of 189.2 μg/mL (MCF‐7), > 200 μg/mL (MDA‐MB‐231) and 173.4 μg/mL (4 T1). Among the compounds isolated from *T. ivorensis* , lupeol (**1**), betulinic acid (**2**) and 3,3′,4′‐Tri‐O‐methylellagic acid‐4‐O‐β‐Dglucopyranoside (**5**) exhibited moderate cytotoxicity on the tested cell lines, ranging from 37.3 μg/mL on the 4 T1 cells for lupeol (**1**) to 71.3 μg/mL on the MDA‐MB‐231 cells for 3,3′,4′‐Tri‐O‐methylellagic acid‐4‐O‐β‐Dglucopyranoside (**5**).

**TABLE 1:** CC_50_ values of *Terminalia ivorensis* crude extract and its isolates on breast cancer cell lines.

Compound	CC_50_ (μg/mL)		
	MCF‐7	MDA‐MB‐231	4 T1
(**1**)	42.1	52.4	37.3
(**2**)	56.4	63.1	49.8
(**3**)	**22.5**	**25.4**	**18.3**
(**4**)	> 100	> 100	> 100
(**5**)	64.5	71.3	58.7
Doxorubicin	0.6	1.1	0.5
*T. ivorensis*	189.2	> 200	173.4

*Note:* CC_50_ = concentration of compound/extract which results in 50% of cell viability. Bold = compound with a cytotoxicity around 20 µg/mL considered to be highly active [27].

The compound 3,3′‐Di‐O‐methylellagic acid‐4′‐O‐β‐D‐glucopyranoside (**4**) did not induce cytotoxicity up to the higher concentration tested (100 mg/mL). Interestingly, arjunolic acid (**3**) exhibited interesting cytotoxicity against breast cancer cells with CC_50_ values of 22.5 μg/mL (MCF‐7), 25.4 μg/mL (MDA‐MB‐231) and 18.3 μg/mL (4 T1).

### 3.3 Effects of Arjunolic Acid on Body Weight Evolution and Survival Rate of Rats

**FIGURE 2:**
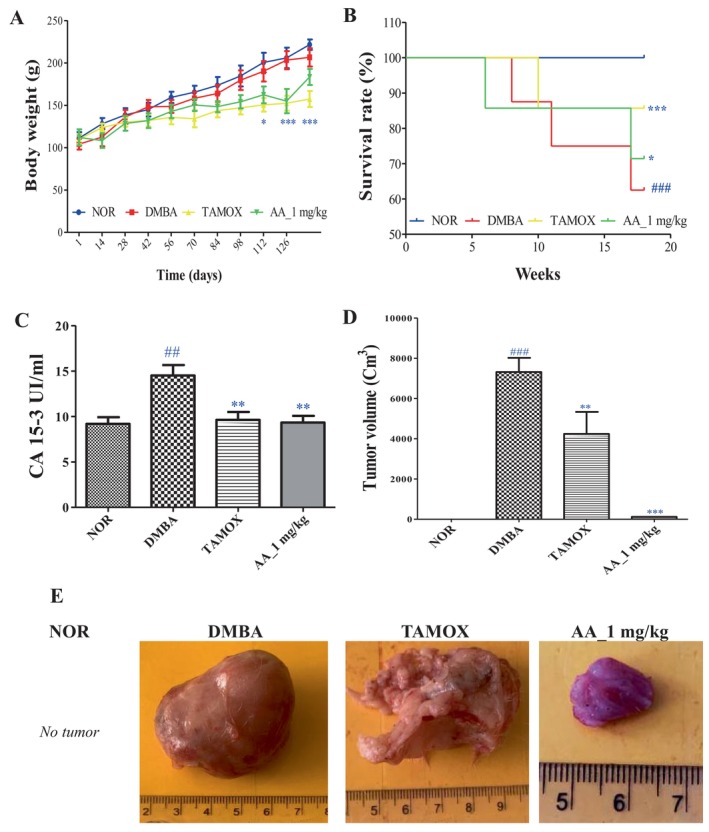
Chemopreventive effect of arjunolic acid on body weight (A), survival rate (B), CA15‐3 level (C), tumor volume (D) and tumor morphology (D). The normal (NOR) and negative control (DMBA) groups that received distilled water (vehicle); Tamox = positive control group treated with tamoxifen at 3.3 mg/kg BW; AA = rats treated with arjunolic acid (**3**) at 1 mg/kg. Points represent means ± SEM (*n* = 8). All rats except those in the normal group received DMBA at a dose of 50 mg/kg. Significance compared to the NOR group: ##*p <* 0.01 ###*p <* 0.001. Significance compared to the DMBA group: *p < 0.05, **p < 0.01, ***p < 0.001.

Figure 2A depicting body weight evolution shows that tamoxifen led to a significant decrease in body weights from day 98 (*p <* 0.05) to day 121 (*p <* 0.001).

Figure 2B depicts the Kaplan–Meier estimate survival curve of rats, on which we can see that no deaths were observed in the normal group (NOR), while the survival percentage in the DMBA group was 60% (*p <* 0.001), followed by the arjunolic acid treated group (AA_1 mg/kg) with 71.62% (*p <* 0.05), and finally the tamoxifen‐treated group (TAMOX) with 85.71% survival (*p <* 0.001).

### 3.4 Effects of Arjunolic Acid on Tumor Parameters

The tumor parameters assessed in this study were tumor incidence, tumor mass, tumor volume, tumor burden, and the percentage inhibition of tumor burden. The normal group did not exhibit any spontaneous breast tumor, while 100% (8/8) of the rats in the DMBA group developed mammary tumors (Table 2).

**TABLE 2:** Effect of Arjunolic acid (**3**) on some breast tumor's parameters.

	Nbr of rats with tumors	Tumor incidence	Tumor mass (mg/kg)	Tumor charge (g/kg)	Inhibition of tumor charge
NOR	0/8	0%	—	—	—
DMBA	8/8	100%	20.28 ± 4.25	96.17 ± 17.17	—
TAMOX	3/8	37.5%	7.09 ± 2.18***	51.01 ± 1.32**	44%
AA_1 mg/kg	1/8	12.5%	1.16 ± 0.10***	5.95 ± 0.00***	89%

*Note:* The normal (NOR) and negative control (DMBA) groups that received distilled water (vehicle); Tamox = positive control group treated with tamoxifen at 3.3 mg/kg BW; AA = rats treated with arjunolic acid (**3**) at 1 mg/kg. Significance compared to DMBA: **p < 0.01, ***p < 0.001.

Table 2 also shows that tamoxifen treatment reduced tumor incidence (37.5%), tumor mass (*p <* 0.001), and induced a 44% inhibition of tumor burden compared to the DMBA group. Similar to tamoxifen, the arjunolic acid treatment reduced tumor incidence (12.5%) and tumor mass and tumor burden inhibition (*p <* 0.001) to a more pronounced extent.

Regarding CA15‐3 levels (Figure 2C), tumor volume (Figure 2D) and tumor morphology (Figure 2E), animals in the DMBA group had the largest tumors and a significantly elevated CA15‐3 level (*p <* 0.01) compared to the normal group. Tamoxifen treatment resulted in a significant decrease in CA15‐3 levels (*p <* 0.01) and tumor volume (*p <* 0.01). Similarly, to tamoxifen, the arjunolic acid induced a significant decrease in serum CA15‐3 levels (*p <* 0.01) and tumor volume (*p <* 0.001).

### 3.5 Effects of Arjunolic Acid on Tumor Microarchitecture

**FIGURE 3:**
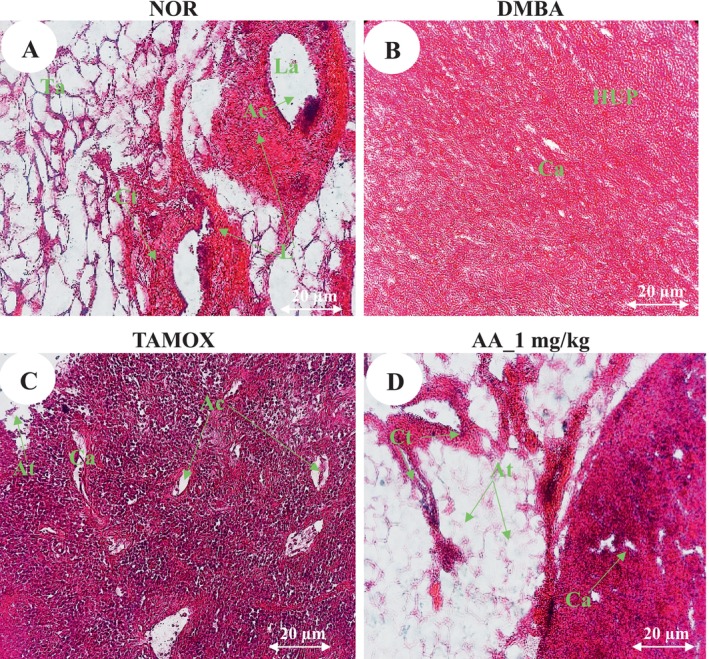
Histopathology of mammary tumors and mammary glands after121 days of treatment with arjunolic acid. The normal (NOR) and negative control (DMBA) groups that received distilled water (vehicle); Tamox = positive control group treated with tamoxifen at 3.3 mg/kg BW; AA = rats treated with arjunolic acid (**3**) at 1 mg/kg. All rats except those in the normal group received DMBA at a dose of 50 mg/kg. At, adipose tissue; Ac, Acinar cells; Cat, cellular atypia; Ct, connective tissue; Hup, High undifferentiated parenchyma; La, Light of the acini; L, lobule; Ne, Necrosis.

Figure 3 illustrates the effects of arjunolic acid on the microarchitecture of mammary tissue. Rats in the normal group (Figure 3A) showed a typical mammary parenchyma composed of numerous lobules of adipocytes surrounding connective tissue strands, from which emerged galactophoric ducts of various calibers. The glandular epithelium was well differentiated and consistently accompanied by a layer of epithelial cells. In contrast, the mammary parenchyma of rats in the DMBA group (Figure 3B) was characterized by tumor proliferation composed of compact clusters of cells showing varying degrees of anisocytosis and anisokaryosis, with both round and spindle‐shaped appearances. Mammary tumor sections from the tamoxifen‐treated group (Figure 3C) showed ongoing tumor proliferation. Tumor cells, either rounded or spindle‐shaped, exhibited marked anisocytosis and anisokaryosis. These cells were arranged either in compact clusters or dissociated within a connective stroma that appeared either dense, edematous, or entirely necrotic. Compared to the DMBA group, cellular elements were more dissociated in some areas, and adipocytes were still present. Sections from tumors in rats treated with arjunolic acid (Figure 3D) revealed a mammary parenchyma infiltrated by tumor cells arranged in sheets and clusters, partially dissociated by edema and replaced by extensive necrotic areas. However, we can observe less differentiation than that of the structure of the DMBA group with the persistence of connective tissues and dense lobules of adipocytes.

### 3.6 Effects of Arjunolic Acid on Serum Levels of Some Cytokines

**FIGURE 4:**
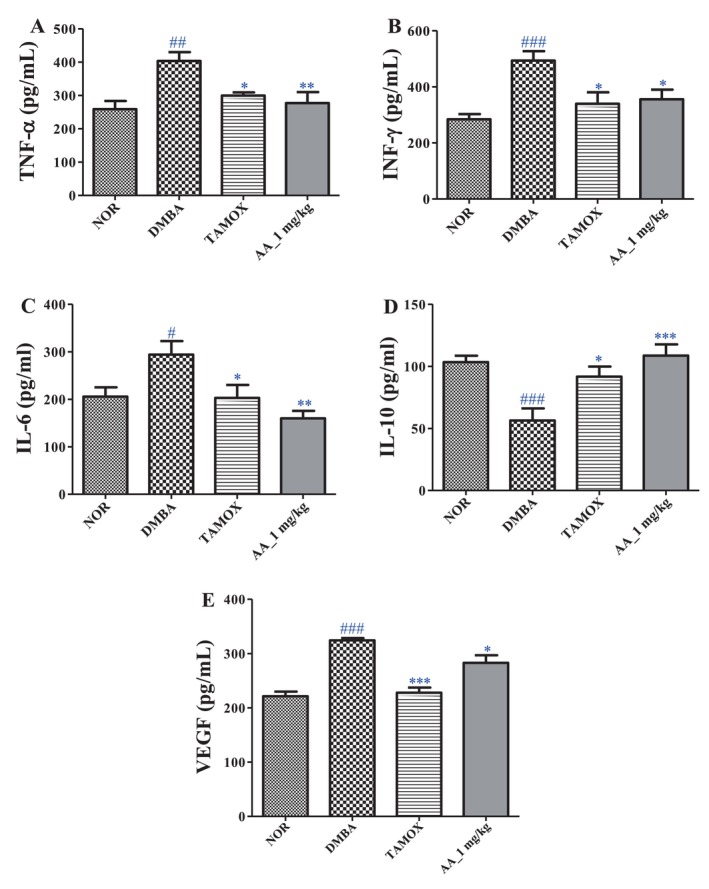
Effects of arjunolic acid on TNF‐α (A), INF‐γ (B), IL‐6 (C), IL‐10 (D) and VEGF (E) levels. The normal (NOR) and negative control (DMBA) groups that received distilled water (vehicle); Tamox = positive control group treated with tamoxifen at 3.3 mg/kg BW; AA = rats treated with arjunolic acid (**3**) at 1 mg/kg. Points represent means ± SEM (*n* = 8). All rats except those the normal group received DMBA at a dose of 50 mg/kg. Significance compared to the NOR group: #*p <* 0.05; ##*p <* 0.01; ###*p <* 0.001. Significance compared to the DMBA group: *p < 0.05, **p < 0.01, ***p < 0.001.

Figure 4 illustrates that DMBA administration caused a significant elevation in serum concentrations of TNF‐α (*p <* 0.01), IFN‐γ (*p <* 0.001), IL‐6 (*p <* 0.05), and VEGF (*p <* 0.001), alongside a marked reduction in IL‐10 levels (*p <* 0.001). Treatment with tamoxifen effectively counteracted these changes, significantly lowering serum levels of TNF‐α (*p <* 0.05), IFN‐γ (*p <* 0.05), IL‐6 (*p <* 0.05), and VEGF (*p <* 0.001), while significantly elevating IL‐10 (*p <* 0.05). Comparable immunomodulatory effects were observed following administration of arjunolic acid, which significantly decreased serum TNF‐α (*p <* 0.01), IFN‐γ (*p <* 0.05), IL‐6 (*p <* 0.01), and VEGF (*p <* 0.05) levels, and notably increased IL‐10 concentrations (*p <* 0.001).

### 3.7 Effects of Arjunolic Acid on Some Hematological Parameters and Relative Organ Mass

Hematological Analyses (Table 3) revealed a marked increase in white blood cell (WBC) count in the DMBA group compared to controls (22.48 ± 0.07 × 10^3^/μL vs. 8.04 ± 0.5 × 10^3^/μL, *p <* 0.001). This was accompanied by a significant rise in the proportions of monocytes (7.45% ± 0.07% vs. 0.86% ± 0.28%, *p <* 0.001) and neutrophils (54.75% ± 1.37% vs. 15.28% ± 1.82%, *p <* 0.001), alongside a notable decrease in lymphocytes (34.47% ± 0.36% vs. 81.30% ± 1.44%, *p <* 0.001). Treatment with arjunolic acid significantly reduced WBC count (*p <* 0.001), increased lymphocyte levels (*p <* 0.001), and lowered granulocyte and monocyte percentages (*p <* 0.001) relative to the DMBA group.

**TABLE 3:** Effect of the treatment with Arjunolic acid on haematological parameters and organ wet weights.

Items	NOR	DMBA	TAMOX	AA_1 mg/kg
Hematological parameters				
WBC	8.1 ± 0.5	22.5 ± 0.1###	7.9 ± 1.2***	5.7 ± 0.8***
LYMP	81.3 ± 1.4	34.5 ± 0.4###	77.6 ± 0.9***	71.3 ± 4.3***
GRAN	15.3 ± 1.8	54.7 ± 1.4###	16.3 ± 1.7***	25.2 ± 3.9***
MONO	0.9 ± 0.3	7.5 ± 0.1###	7.9 ± 0.3	3.1 ± 0.6***
RBC	7.1 ± 0.1	5.1 ± 0.2##	6.4 ± 0.8	6.9 ± 0.5
HGB	13.9 ± 0.1	10.2 ± 0.1#	11.8 ± 1.6	12.5 ± 0.5
HCT	42.2 ± 1.1	33.2 ± 1.1#	41.9 ± 2.9*	41.9 ± 1.9*
PLT	565.5 ± 17.4	1070.8 ± 0.1###	801.7 ± 4.5***	618.5 ± 30.2***
Organ wet weight (mg/kg)				
Heart	2815.2 ± 121.7	2813.5 ± 169.7	3490.7 ± 351.1	3179.3 ± 256.6
Uterine	2528.9 ± 61.7	2144.9 ± 181.3###	773.4 ± 61.7***	3448.9 ± 354.5***
Liver	28128.3 ± 1549.2	35604.6 ± 1483.4##	29829.2 ± 1960.3*	24214.9 ± 1831.3***
Kidneys	4218.2 ± 167.8	6102.8 ± 224.3###	6612.3 ± 415.2	5444.8 ± 439.6
Adrenals	202.1 ± 12.8	310.5 ± 20.0###	266.2 ± 18.3	285.7 ± 57.4
Lungs	8172.4 ± 421.6	7314.9 ± 454.5	7574.4 ± 718.1	7720.9 ± 669.2
Femur	2777.2 ± 140.5	2476.4 ± 131.6	3351.7 ± 227.9*	3351.6 ± 367.1*
Spleen	2924.5 ± 133.1	6447.9 ± 378.1###	4604.6 ± 266.1*	4049.2 ± 555.1**
Brain	5242.9 ± 312.6	8294.1 ± 285.9###	7475.5 ± 284.2	6060.81 ± 234.66*
Ovaries	537.1 ± 40.8	554.6 ± 18.5	176.2 ± 22.5**	500.8 ± 127.4

*Note:* The normal (NOR) and negative control (DMBA) groups that received distilled water (vehicle); Tamox = positive control group treated with tamoxifen at 3.3 mg/kg BW; AA = rats treated with arjunolic acid (**3**) at 1 mg/kg. Significance compared to NOR: #*p* < 0.05, ##*p* < 0.01, ###*p* < 0.001; Significance compared to DMBA: *p < 0.05, **p < 0.01, ***p < 0.001.

The DMBA‐treated rats also exhibited significantly decreased red blood cell (RBC) counts (5.04 ± 0.17 × 10^3^/μL vs. 7.14 ± 0.09 × 10^3^/μL, *p <* 0.001), hemoglobin (HGB) levels (*p <* 0.05), hematocrit (HCT) (33.16% ± 1.03% vs. 42.14% ± 0.09%, *p <* 0.001), and platelet counts (1070.8 ± 0.03 × 10^3^/μL vs. 565.5 ± 17.37 × 10^3^/μL, *p <* 0.001), although the decrease in hemoglobin did not reach statistical significance. Compared with the DMBA group, arjunolic acid significantly improved RBC counts (*p <* 0.01) and HCT (*p <* 0.05), while significantly reducing platelet counts (*p <* 0.001). These effects were largely mirrored by tamoxifen treatment, which also significantly decreased WBC, granulocyte, monocyte, and platelet counts (*p <* 0.001), and elevated HCT (*p <* 0.05) and lymphocyte counts (*p <* 0.001).

Relative Organ Mass (Table 3) analysis showed that DMBA caused significant increases in the relative weights of the liver (*p <* 0.01), kidneys, spleen, adrenal glands, and brain (all *p <* 0.001), along with a significant reduction in uterine mass (*p <* 0.001). Tamoxifen administration (3.3 mg/kg) led to significant decreases in the relative weights of the uterus (*p <* 0.001), liver (*p <* 0.05), spleen (*p <* 0.05), and ovaries (*p <* 0.05). In contrast, arjunolic acid treatment significantly increased uterine (*p <* 0.001) and femoral (*p <* 0.05) relative mass, while reducing liver (*p <* 0.001), spleen (*p <* 0.01), and brain (*p <* 0.05) weights.

### 3.8 Effects of Arjunolic Acid on Liver and Kidney Function Markers

**FIGURE 5:**
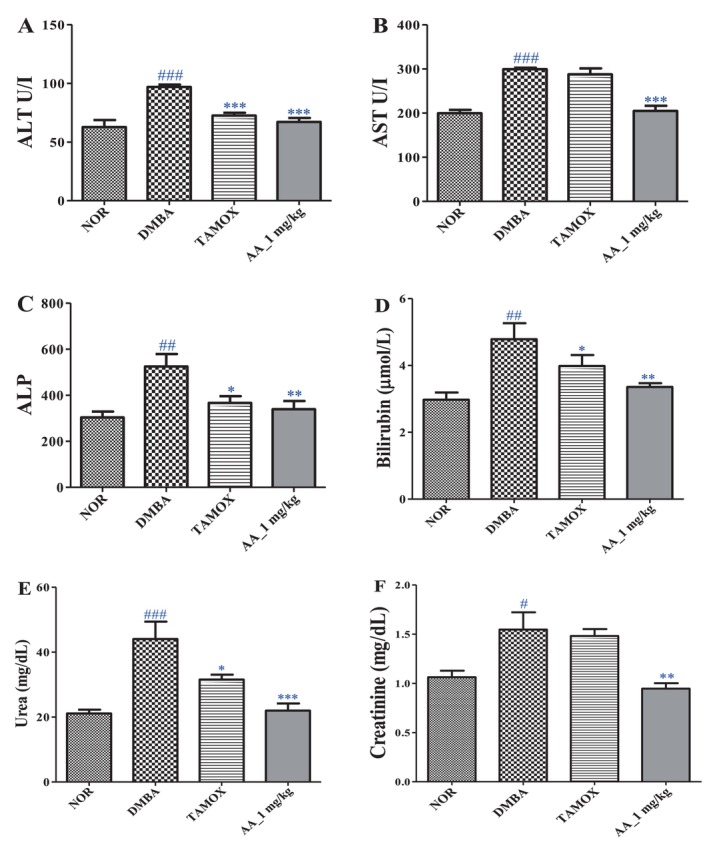
Effects of arjunolic acid on ALT (A), AST (B) and ALP (C) activities as well as bilirubin (D), urea (E) and creatinine (F) levels. The normal (NOR) and negative control (DMBA) groups that received distilled water (vehicle); Tamox = positive control group treated with tamoxifen at 3.3 mg/kg BW; AA = rats treated with arjunolic acid (**3**) at 1 mg/kg. Points represent means ± SEM (*n* = 8). All rats except those the normal group received DMBA at a dose of 50 mg/kg. Significance compared to the NOR group: #*p <* 0.05; ##*p <* 0.01; ###*p <* 0.001. Significance compared to the DMBA group: *p < 0.05, **p < 0.01, ***p < 0.001.

Relative to the normal group, DMBA administration resulted in significant elevations in serum alanine aminotransferase (ALT) and aspartate aminotransferase (AST) activities (*p <* 0.001), alkaline phosphatase (ALP) levels (*p <* 0.001), and total bilirubin (*p <* 0.01) (Figure 5). Serum urea (*p <* 0.001) and creatinine (*p <* 0.05) concentrations were also significantly increased (Figure 5E,F). Treatment with tamoxifen significantly reduced ALT (*p <* 0.001), ALP (*p <* 0.05), and urea (*p <* 0.05) levels. Similarly, arjunolic acid significantly decreased ALT and AST activities (*p <* 0.001), ALP levels (*p <* 0.01), serum urea (*p <* 0.001), bilirubin (*p <* 0.05), and creatinine (*p <* 0.01) compared to the DMBA group (Figure 5).

## 4 Discussion

Cancer is a multifaceted genetic disorder characterized by unregulated cell proliferation and abnormal growth, often leading to metastasis in distant tissues [28]. Although numerous therapeutic options exist for breast cancer, optimal outcomes remain elusive due to systemic toxicity, drug resistance, and adverse side effects [29]. Consequently, the development of novel anticancer compounds that offer improved efficacy is an urgent priority [7]. In this context, natural products represent a promising source of new anticancer agents, as approximately 60% of anticancer drugs currently employed in the United States are derived from natural origins [30]. Accordingly, this study was designed to investigate the anticancer potential of *T. ivorensis* extracts and isolated compounds through MTT assays and a DMBA‐induced breast cancer rat model.

In the art of screening for anticancer substances, the main strategy is to test the substances for their potential to inhibit tumor growth. The MTT assay is one of the highly used bioassays to test cell growth since it measures the drug's capacity to inhibit cell growth by altering cellular functions like cell mitochondrial function [31]. In this study, *T. ivorensis* methanolic extract exhibited cytotoxicity on the 3 tested cell lines with CC_50_ values of 189.2, > 200, and 173.4 for MCF‐7, MDA‐MB‐231, and 4 T1 cells, respectively. According to established standards in the search for new cytotoxic substances, a threshold CC_50_ value of 4 μg/mL (or 10 μM) is sought for pure compounds and 20 μg/mL for crude extract after 48 and 72 h of incubation [8, 27]. However, this result is of interest because it is the first report on the cytotoxicity of *T. ivorensis* on breast cancer cells. The only data available on the cytotoxicity of this plant is those reported with the ethanolic stem bark extract (close in polarity to the methanolic extract used in this work) on colon cancer (Caco‐2) and non‐malignantly transformed human colonocyte (NCM460) cells, where it displayed a CC_50_ of 3.99 mg/mL and 6.74 mg/mL, respectively [32]. These results support our view that *T. ivorensis* has a low cytotoxic potential. Among the compounds isolated from *T. ivorensis* , lupeol (**1**) and betulinic acid (**2**) exhibited cytotoxicity with an average CC_50_ of ~44 and 56.4 μg/mL, respectively. Lupeol (**1**) and betulinic acid (**2**) are well known for their interesting anti‐cancer properties against a panel of cancer cells that are sensitive or resistant to conventional drugs by multiple mechanisms, with little or no harmful effects on normal cells at similar concentration [33, 34, 35, 36]. The compound 3,3′‐Di‐O‐methylellagic acid‐4′‐O‐β‐D‐glucopyranoside (**4**) did not induce cytotoxicity up to 100 mg/mL, while 3,3′,4′‐Tri‐O‐methylellagic acid‐4‐O‐β‐D‐glucopyranoside (**5**) exhibited moderate cytotoxicity on the tested cell lines with an average CC_50_ of ~64.8 μg/mL. No report on the cytotoxicity of compound (**5**) has been found in the literature, but Lantovololona et al. [24] showed that it possesses antioxidant properties (IC_50_ = 63.9 ± 0.1 μg/mL) and a toxic effect (mortality rate of 65%) toward brine shrimp larvae ( *Artemia salina* ) at 10 μg/mL. In an analysis of the structure–activity relationship, it is interesting to note that the addition of a methyl group to ellagic acid glucoside confers cytotoxicity on compound (**5**) compared with compound (**4**). As far as arjunolic acid (**3**) is concerned, it can be noted on its ^13^C NMR spectrum the presence of some small peaks around ~138 ppm and ~125 ppm, characteristic of asiatic acid [37]. This is very common in the purification of triterpenoid saponins, as many authors have pointed out. Given the very low proportion of asiatic acid, the effects observed are entirely attributable to arjunolic acid. Arjunolic acid (**3**) exhibited an interesting cytotoxicity effect against breast cancer cells with CC_50_ values of 22.5 μg/mL (MCF‐7), 25.4 μg/mL (MDA‐MB‐231) and 18.3 μg/mL (4 T1) in this study. In fact, it is a triterpenoid saponin initially isolated from the bark of *Terminalia Arjuna*, which is endowed with many pharmacological activities among which those in line with the observed cytotoxicity are antifungal [38], antibacterial [39] and cytotoxicity [40]. With respect to cell death, its effect against lung adenocarcinoma A549, Ehrlich ascites carcinoma, and Dalton's lymphoma cell lines were 82%, 66%, and 70%, respectively, at 100 μg/mL concentration; indicating its ability to disrupt the membrane of cancer cells [40, 41].

To assess the chemopreventive potential of arjunolic acid, the well‐characterized model of breast cancer induced in female rats by DMBA was used. This model is highly acclaimed because it reproduces in many respects the pathogenesis of mammary carcinogenesis in humans at morphological, histological, biochemical, and molecular levels [25, 42]. It is also interesting in that the carcinogen used is found in the environment and is a potential initiator of cancer in humans. Arjunolic acid improved the survival rate (71.62%) compared to age‐matched rats exposed to DMBA alone (60%), or those exposed to DMBA and treated with tamoxifen (85.71%). This result indicates that arjunolic acid exerts a protective effect against the hemato‐ and immune‐toxicities of DMBA, which have been recognized as responsible for most of its lethality associated with tumor progression. Tumor‐related parameters explored further highlight the antitumor potential of arjunolic acid. Indeed, an 89% inhibition in tumor burden, compared to 44% with tamoxifen, underscores the compound's notable efficacy in controlling tumor growth. This effect may arise from multiple mechanisms, including apoptosis induction, inhibition of cell proliferation, and modulation of the tumor microenvironment. Findings from Gonçalves et al. [43] support the hypothesis that arjunolic acid derivatives target key signaling pathways by inhibiting oncogenic transcription factors and proteins while promoting the activation of antitumor pathways. These results are in accordance with those of Elsherbiny et al. [44], who reported that arjunolic acid abrogated tumor volume in Ehrlich Ascites Carcinoma cells in vitro and in vivo at 100 and 250 mg/kg concentrations through induction of apoptosis. It should be noted that the doses used by these authors are much higher than those used in this study (1 mg/kg), but this can be explained by the fact that these authors tested the therapeutic potential of arjunolic acid in a short period (14 days), whereas we were looking at the chemopreventive potential over a long period (121 days). According to the same authors, part of the anti‐tumor properties of arjunolic acid was due to its capability to modulate inflammatory cytokines. It decreases TNF‐α, IL‐1β, TGF‐β levels, and increased IL‐10 level in Ehrlich Ascites Carcinoma cells in vitro and in vivo [44]. The administration of arjunolic acid in this study led to a significant reduction in pro‐inflammatory cytokines such as TNF‐α, IFN‐γ, and IL‐6, along with lower levels of VEGF (a key factor in tumor angiogenesis) and a marked increase in anti‐inflammatory cytokine IL‐10. This simultaneous modulation, which is in line with those observed by Elsherbiny et al. [44] suggests that arjunolic acid acts both by reducing chronic inflammation and regulating the tumor microenvironment, thus creating a favorable context for an enhanced adaptive immune response. Such a balanced immune response is likely to contribute to better tumor control by limiting cancer cell proliferation and the angiogenesis required for tumor survival.

One of the prerequisites for studying candidate cancer drugs is their safety, and toxicological parameters need to be evaluated. A crucial observation lies in the monitoring of body weight. The stable body weight observed in the arjunolic acid‐treated group, compared to the significant weight loss noted with tamoxifen, known for its ability to inhibit food intake [25], suggests a positive impact of arjunolic acid on nutritional status and overall well‐being. In the context of oncology, stable body weight is an essential indicator of treatment tolerance, particularly in combating cancer cachexia, a syndrome marked by muscle wasting and decreased body mass, contributing to increased morbidity [45]. Another important aspect concerns relative organ weights, considered sensitive indicators of systemic toxicity. DMBA caused a significant increase in the weight of organs such as the liver, kidneys, spleen, adrenals, and brain, while decreasing the relative weight of the uterus. Conversely, arjunolic acid significantly increased the relative mass of the uterus and femur, and reduced the weights of the liver, spleen, and brain. These observations suggest that arjunolic acid may exert protective effects on specific organs by limiting DMBA‐induced damage while preserving reproductive function. Moreover, hematological and biochemical analyses revealed that DMBA induced alterations, including increased white blood cells, neutrophils, and monocytes, alongside decreased lymphocyte counts and impaired liver and kidney function (as shown by elevated ALT, AST, ALP, bilirubin, urea, and creatinine levels). In this context, treatment with arjunolic acid appeared to normalize these parameters, aligning them closer to those observed in the normal group. Notably, improvement in hematocrit and stabilization of several biochemical markers suggest partial protection against DMBA‐induced myelosuppressive and hepatotoxic effects [46]. Our findings correlate with those of Aamir et al. [47], who demonstrated that arjunolic acid is safe and well tolerated at doses of 300 and 2000 mg/kg based on acute oral toxicity assessment done in normal female Sprague Dawley rats. These findings reflect the compound's capacity to mitigate metabolic stress and organ damage linked to carcinogenesis [48]. The safety of arjunolic acid despite its anticancer potential highlights the interest of arjunolic acid as a potential chemopreventive agent. In a context where conventional treatments such as chemotherapy frequently lead to severe side effects, arjunolic acid emerges as a promising candidate for combined or substitute therapies in patients with contraindications to standard care.

## 5 Conclusion

In this study, the potential cytotoxicity of *T. ivorensis* has been explored and its active ingredients isolated. Out of the 5 isolates [lupeol (**1**), betulinic acid (**2**), Arjunolic acid (**3**), 3,3′‐Di‐O‐methylellagic acid‐4′‐O‐β‐D‐glucopyranoside (**4**) and 3,3′,4′‐Tri‐O‐methylellagic acid‐4‐O‐β‐D‐glucopyranoside(**5**)] from *T. ivorensis* , compound (**3**) had the most significant inhibitory effect against breast cancer cell growth. In vivo, a significant improvement in survival, a reduction (~89%) in tumor burden, and favorable modulation of the tumor microenvironment, characterized by a decrease in pro‐inflammatory cytokines (TNF‐α, IFN‐γ, IL‐6, VEGF) and an increase in IL‐10, was observed following treatment with arjunolic acid. We encourage further studies on this compound to better identify its modes and mechanisms of action.

## Author Contributions

Patrick Yves Ango, Muriel Angounou Akamse, Ghislain Wabo Fotso, Marthe Carine Djuidje Fotsing, Edwin Mmutlane, Derek Tantoh Ndinteh, and Deccaux Wabo Fotso Gilbert Kapche realized the phytochemical part of the work and interpreted the NMR data. Vigny Diembo Kamgo and Stephane Zingué participated in the in vitro and in vivo experiments. Vigny Diembo Kamgo, Patrick Yves Ango, Derek Tantoh Ndinteh, Edwin Mmutlane, and Stephane Zingué designed the study, finalized the manuscript, and Deccaux Wabo Fotso Gilbert Kapche supervised the work.

## Conflicts of Interest

The authors declare no conflicts of interest.

## Supporting information


**Data S1:** Supporting Information.

## Data Availability

The data that support the findings of this study are available from the corresponding author upon reasonable request.
