# Supplementary material for: Arjunolic Acid From Terminalia ivorensis A. Chev (Combretaceae) Possesses Anti‐Breast Cancer Effects In Vitro and In Vivo
Source: Cancer Rep (Hoboken). 2025 Sep 4;8(9):e70337. doi: 10.1002/cnr2.70337 (PMC12411669; doi:10.1002/cnr2.70337)
Supplement: Supplementary file 1 — Data S1: Supporting Information. [file CNR2-8-e70337-s001.pdf]

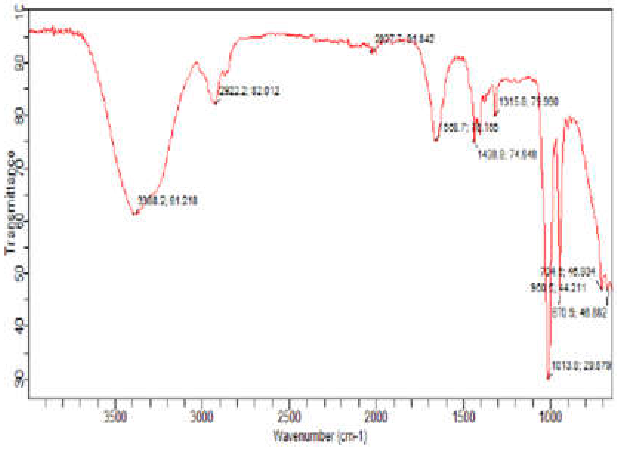

Figure S1: IR spectrum of Lupeol

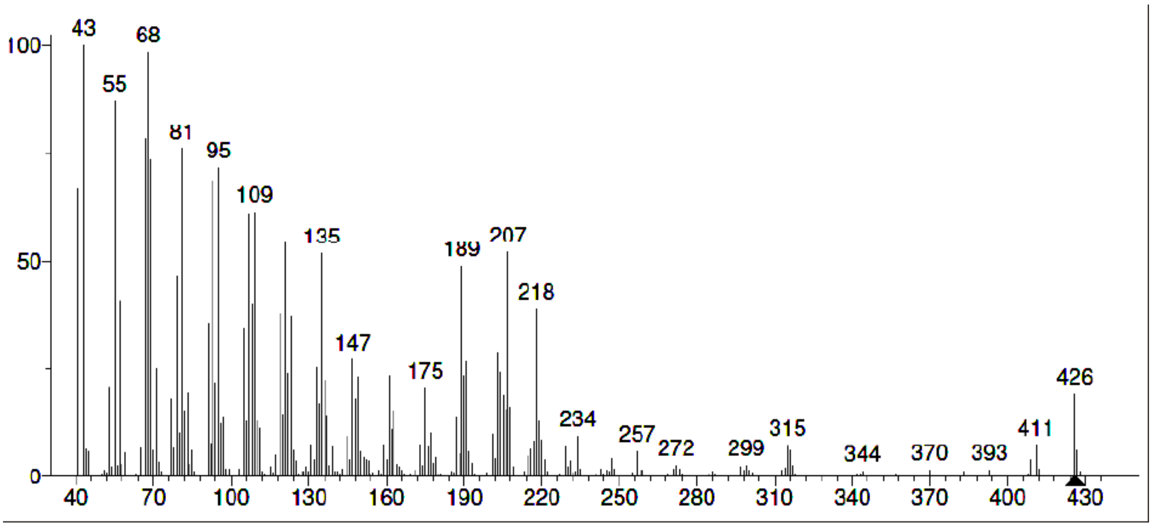

Figure S2: Masse spectrum of Lupeol

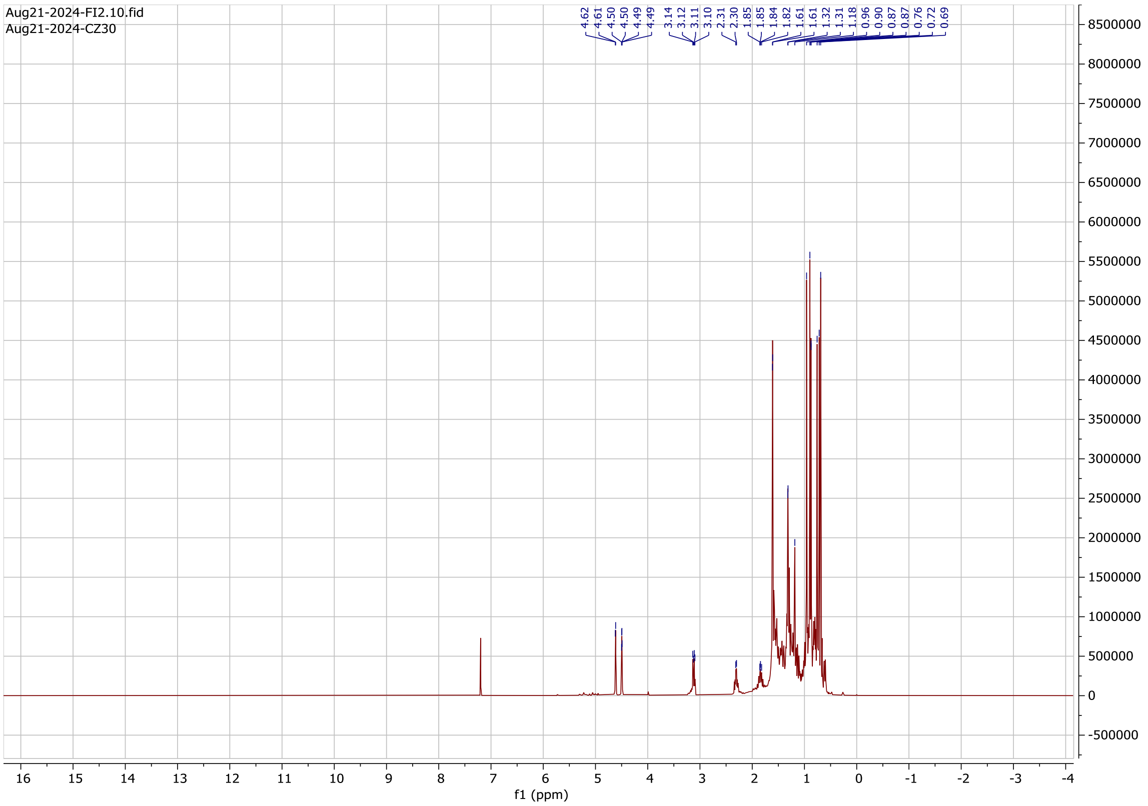

Figure S3: ^{1}H NMR spectrum of Lupeol

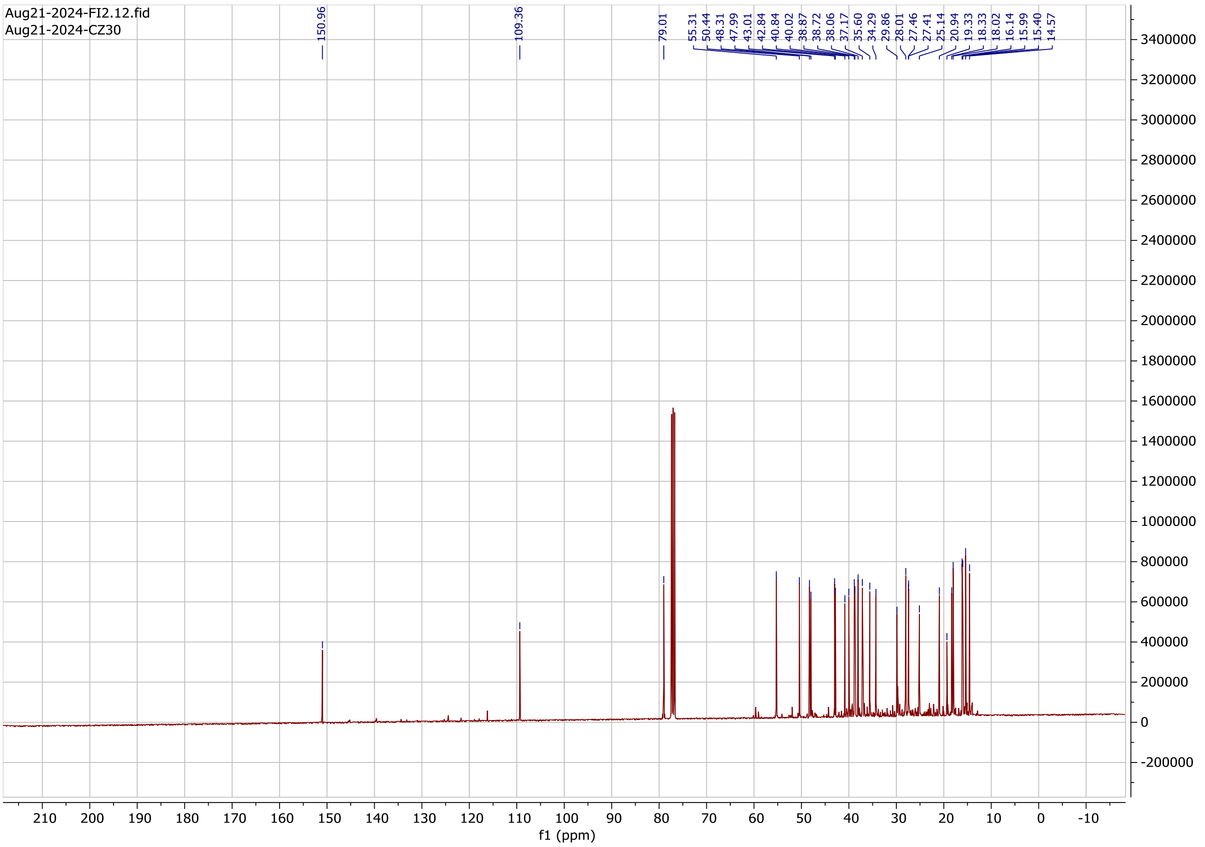

Figure S4: ^{13}C NMR spectrum of Lupeol

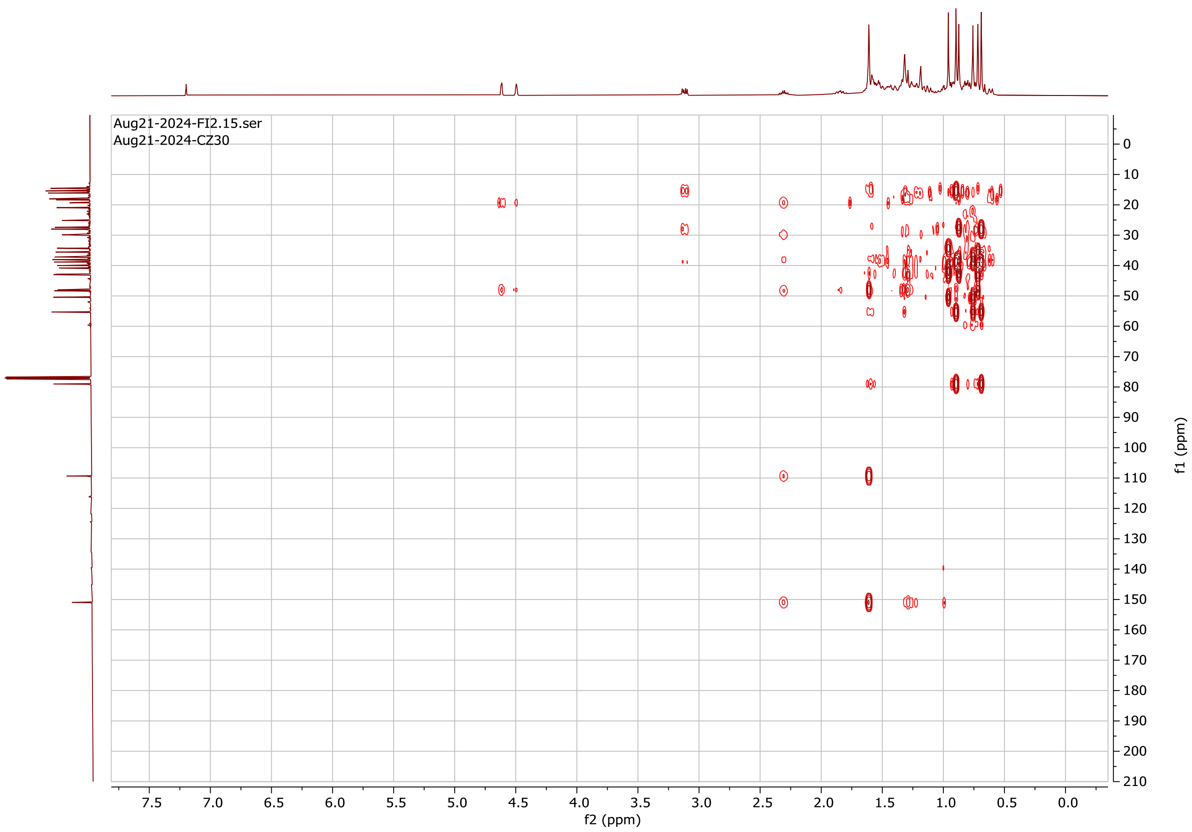

Figure S5: HMBC spectrum of Lupeol

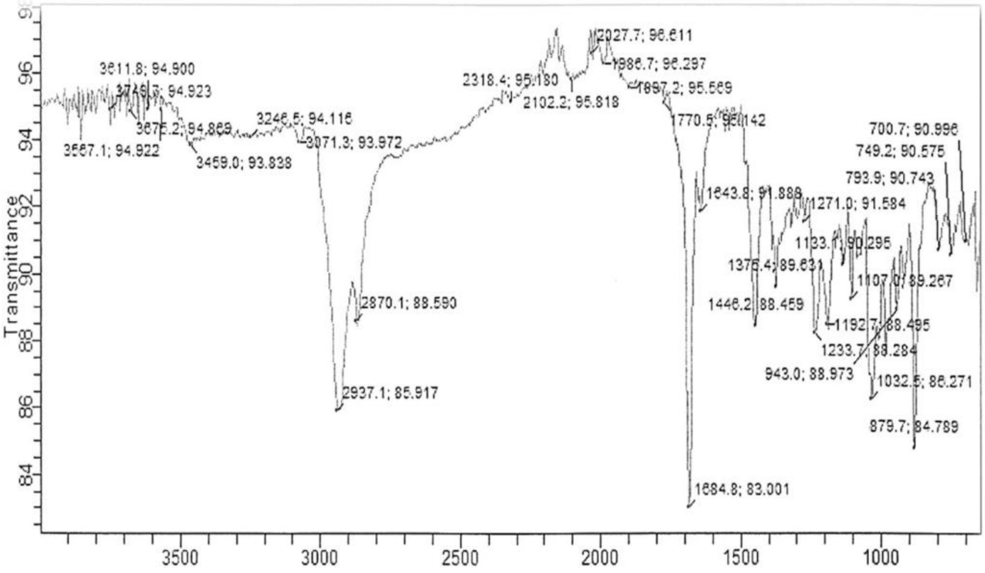

Figure S6: IR spectrum of Betulinic acid

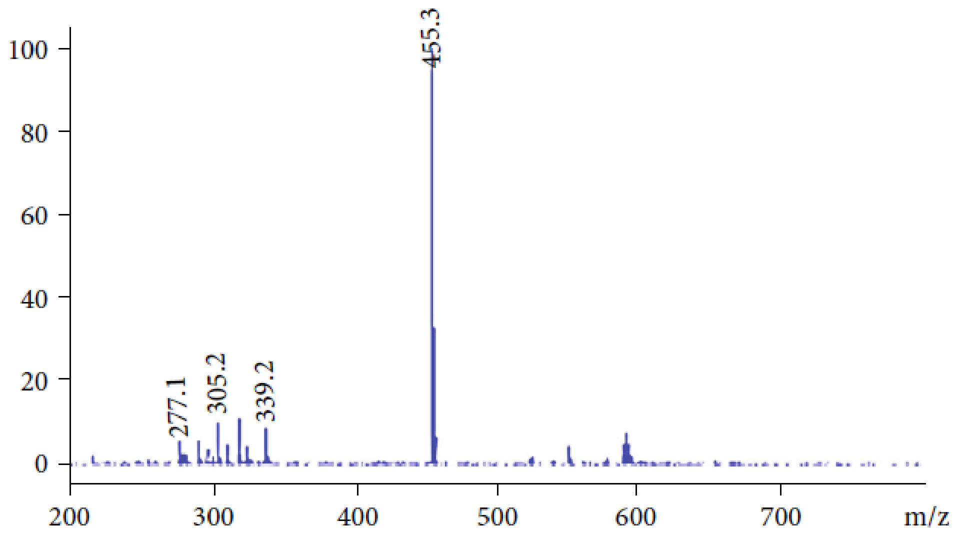

Figure S7: Mass spectrum of Betulinic acid

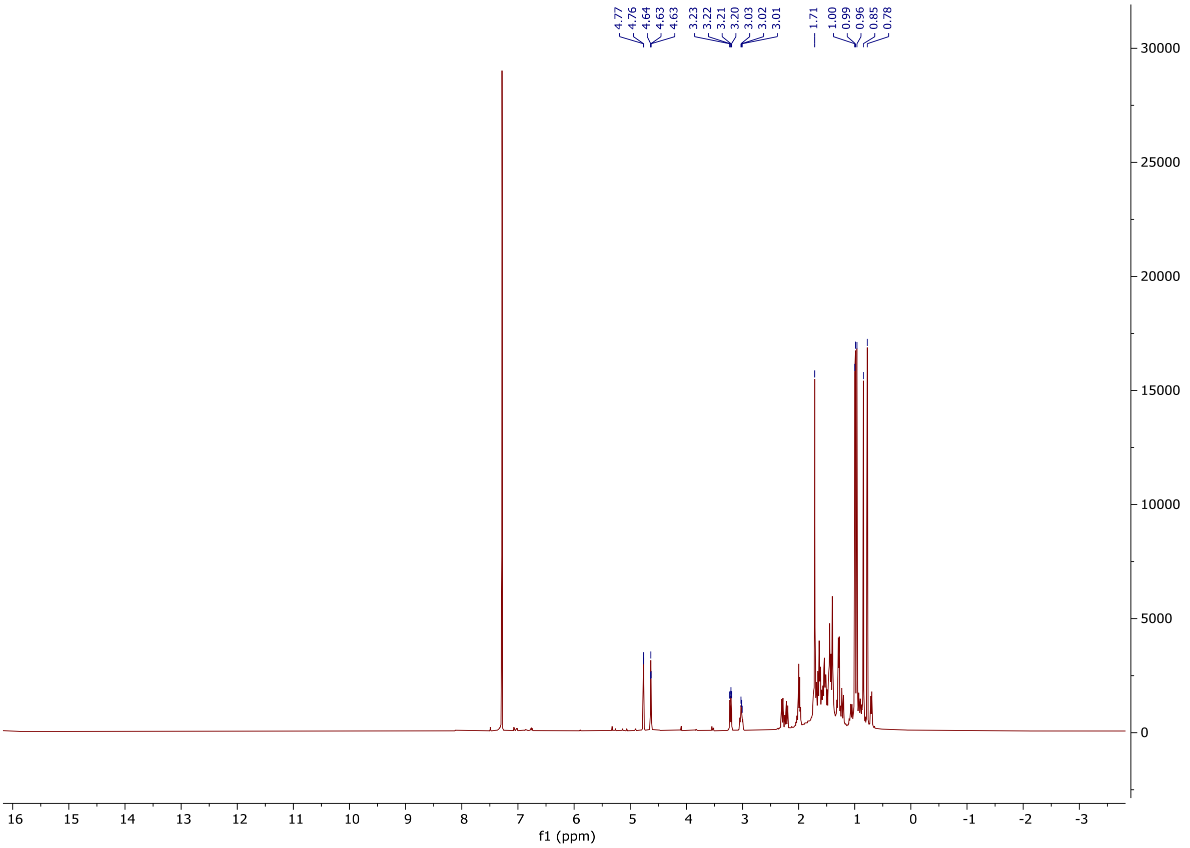

Figure S8: ^{1}H NMR spectrum of Betulinic acid

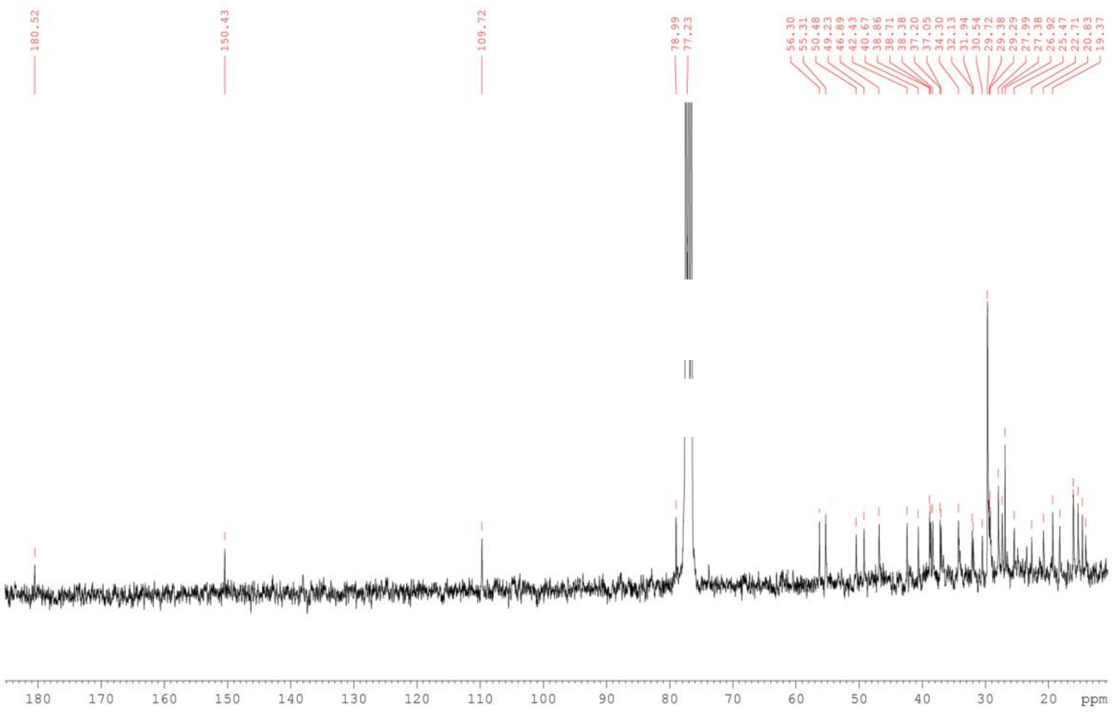

Figure S9: ^{13}C NMR spectrum of Betulinic acid

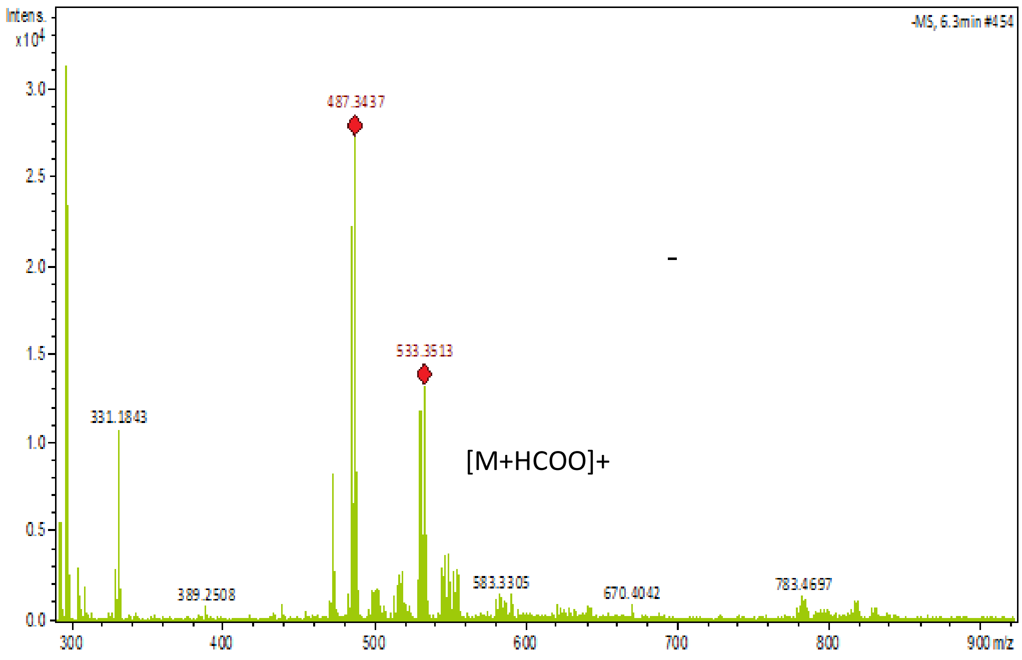

Figure S10: Mass spectrum of Arjunolic acid

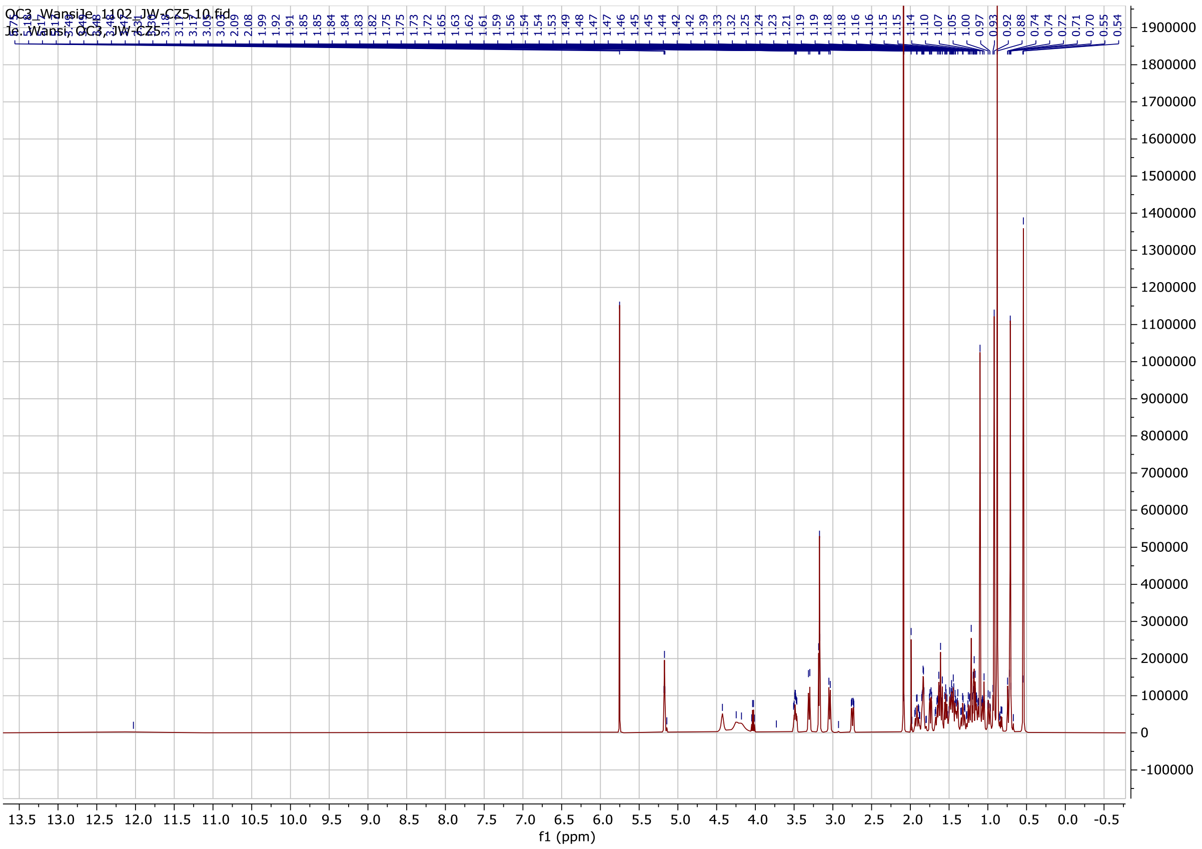

Figure S11: ^{1}H NMR spectrum of Arjunolic acid

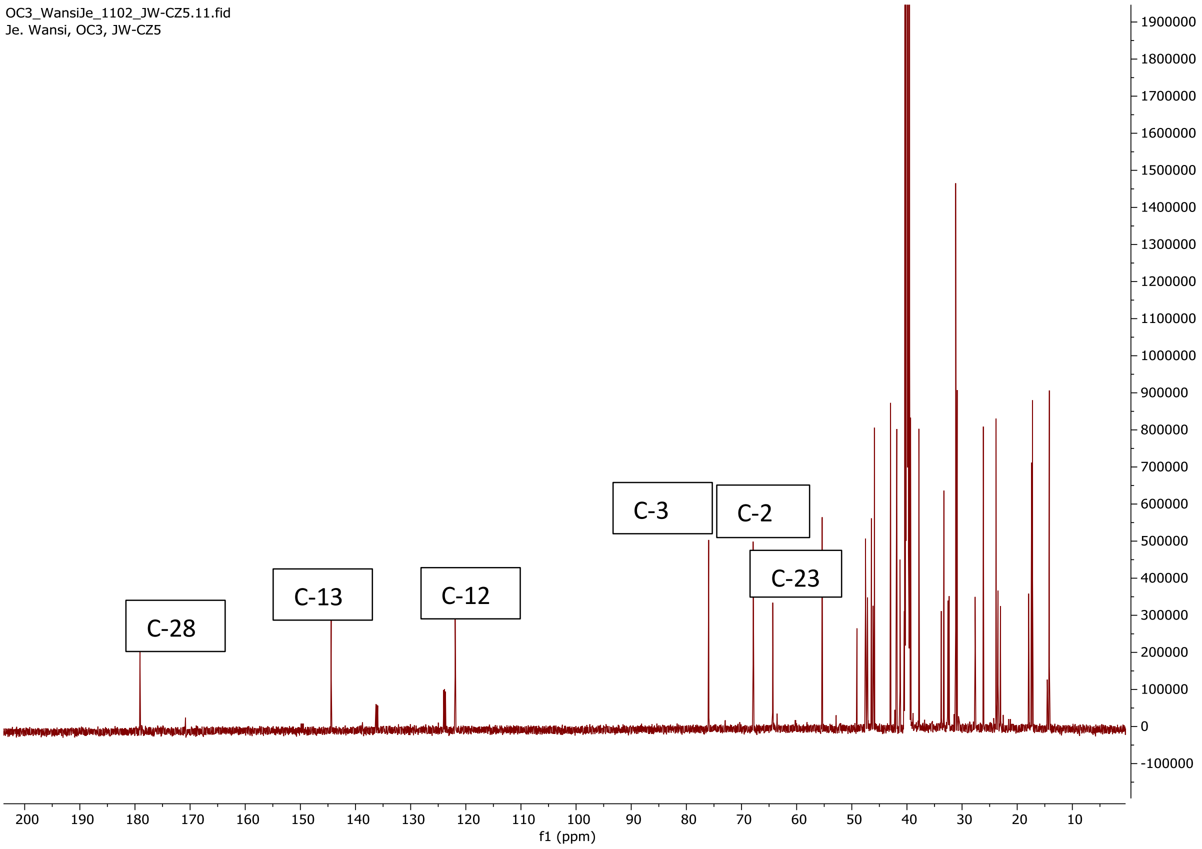

Figure S12: ^{13}C NMR spectrum of Arjunolic acid

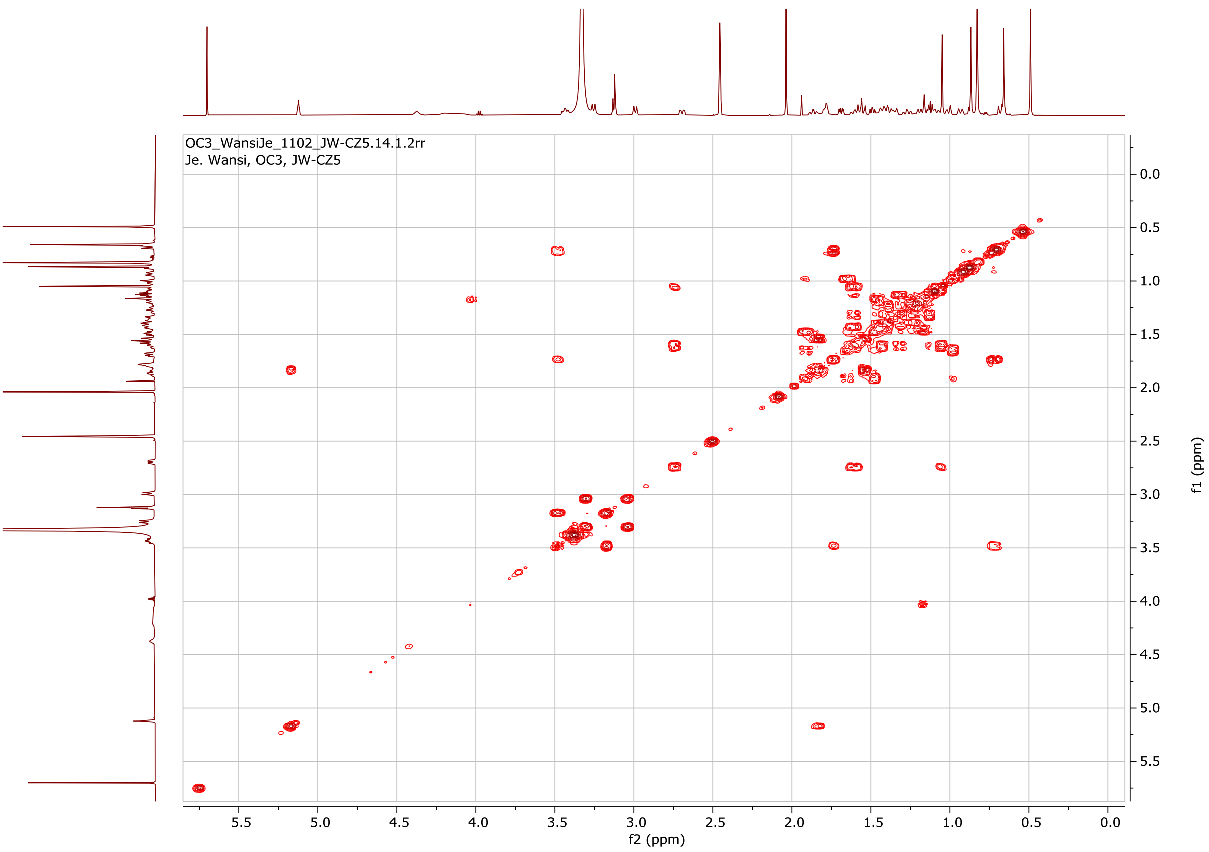

Figure S13: COSY spectrum of Arjunolic acid

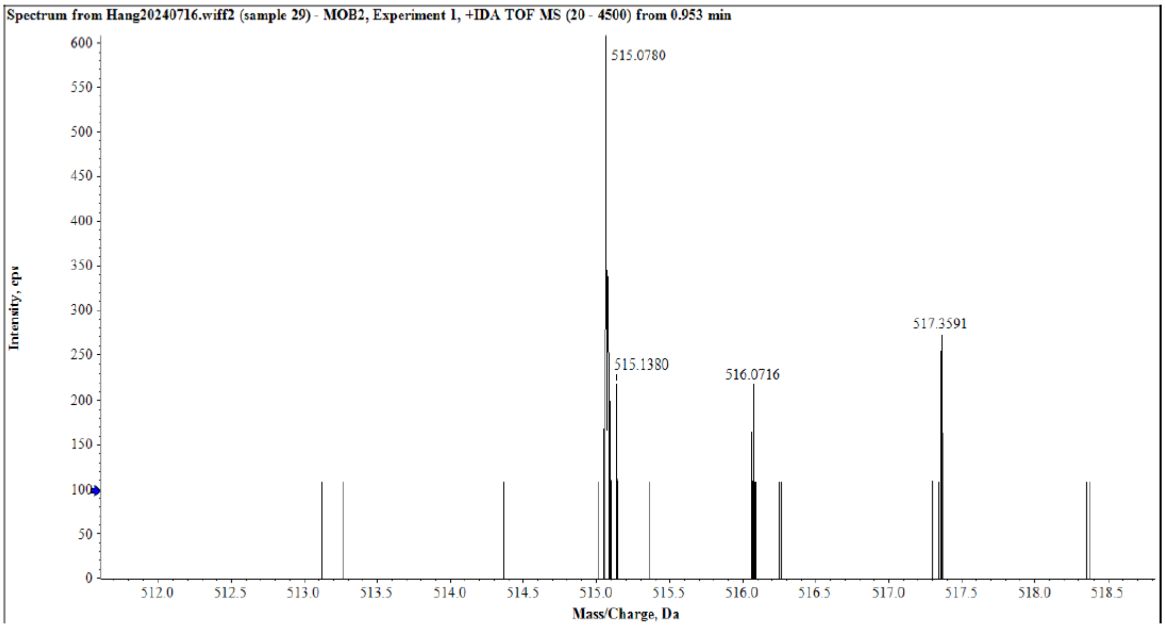

Figure S14: Mass spectrum of 3,3'-Di-O-methylellagic acid-4'-O-β-D-glucopyranoside

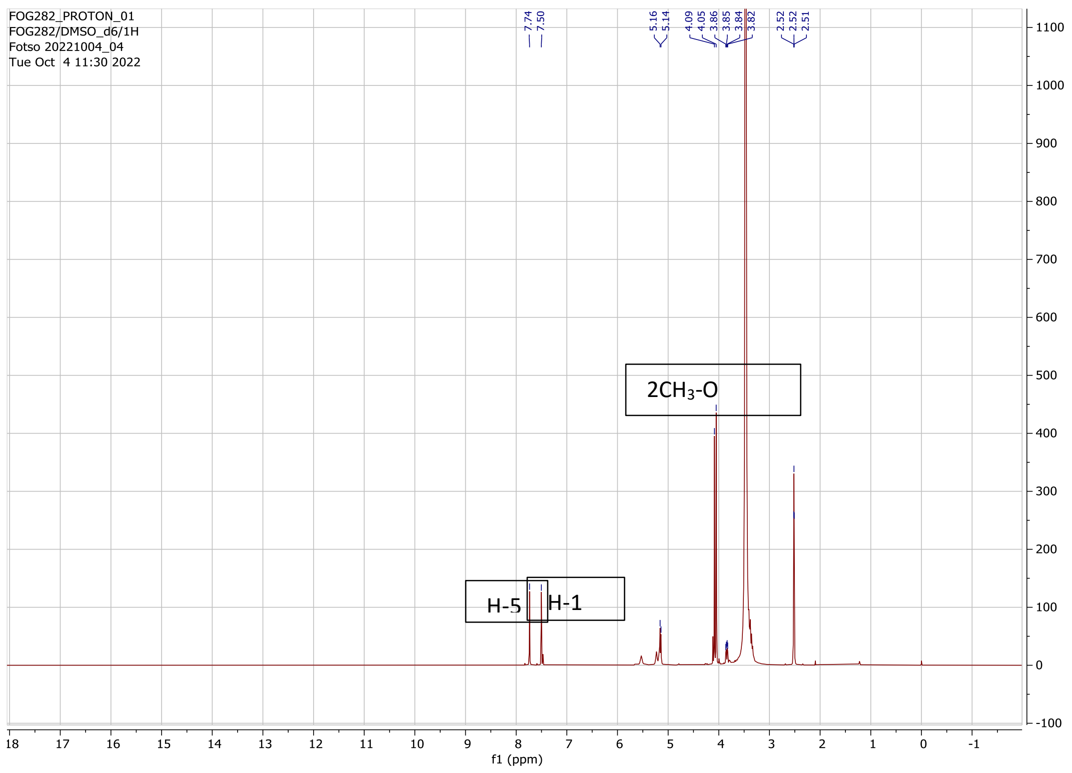

Figure S15: ^{1}H NMR spectrum of 3,3'-Di-O-methylellagic acid-4'-O-β-D-glucopyranoside

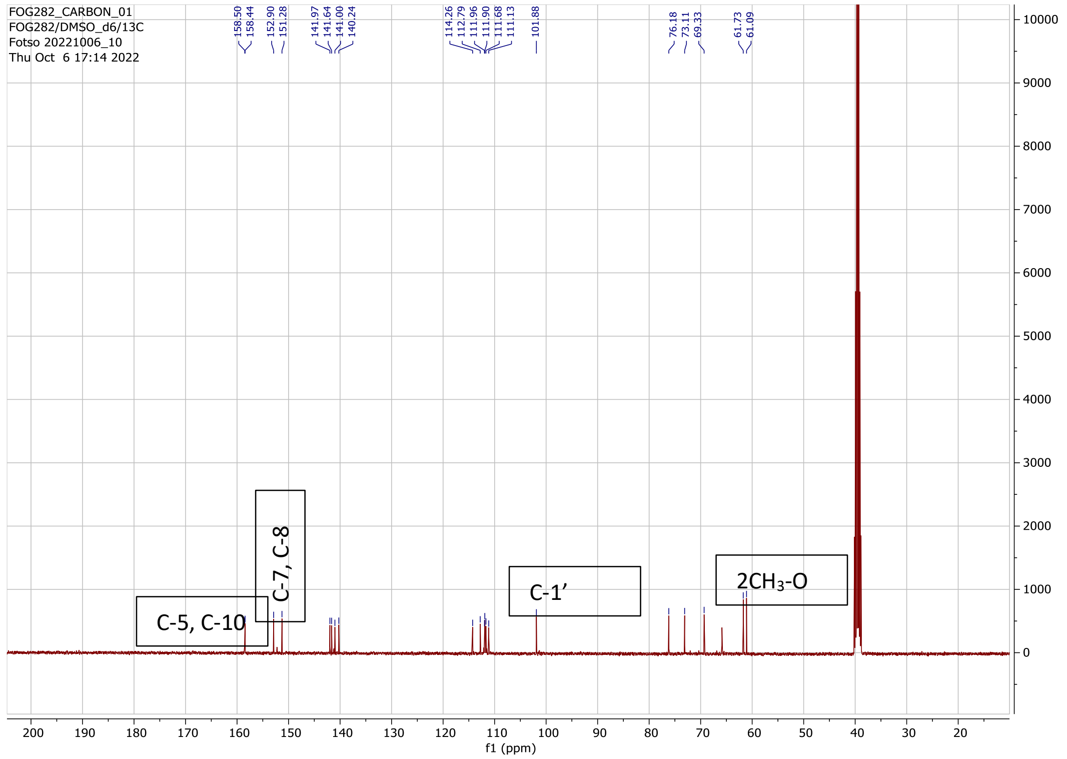

Figure S16: ^{13}C NMR spectrum of 3,3'-Di-O-methylellagic acid-4'-O-β-D-glucopyranoside

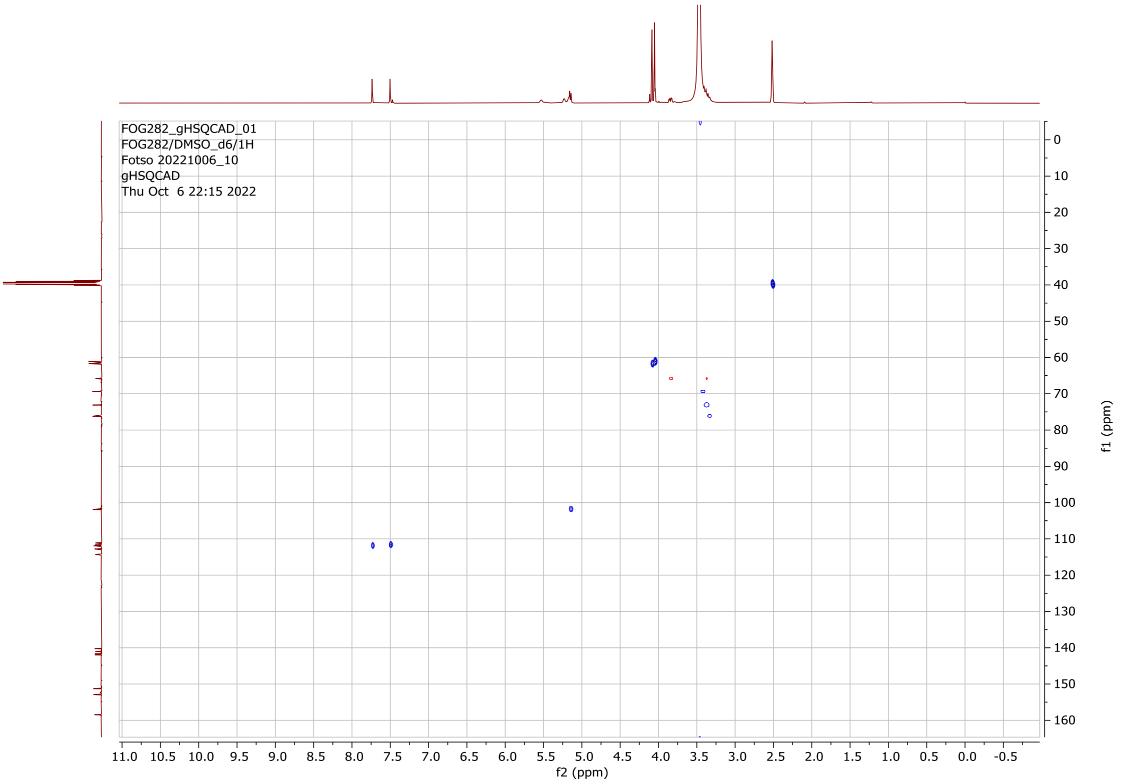

Figure S17: HSQC spectrum of 3,3'-Di-O-methylellagic acid-4'-O-β-D-glucopyranoside

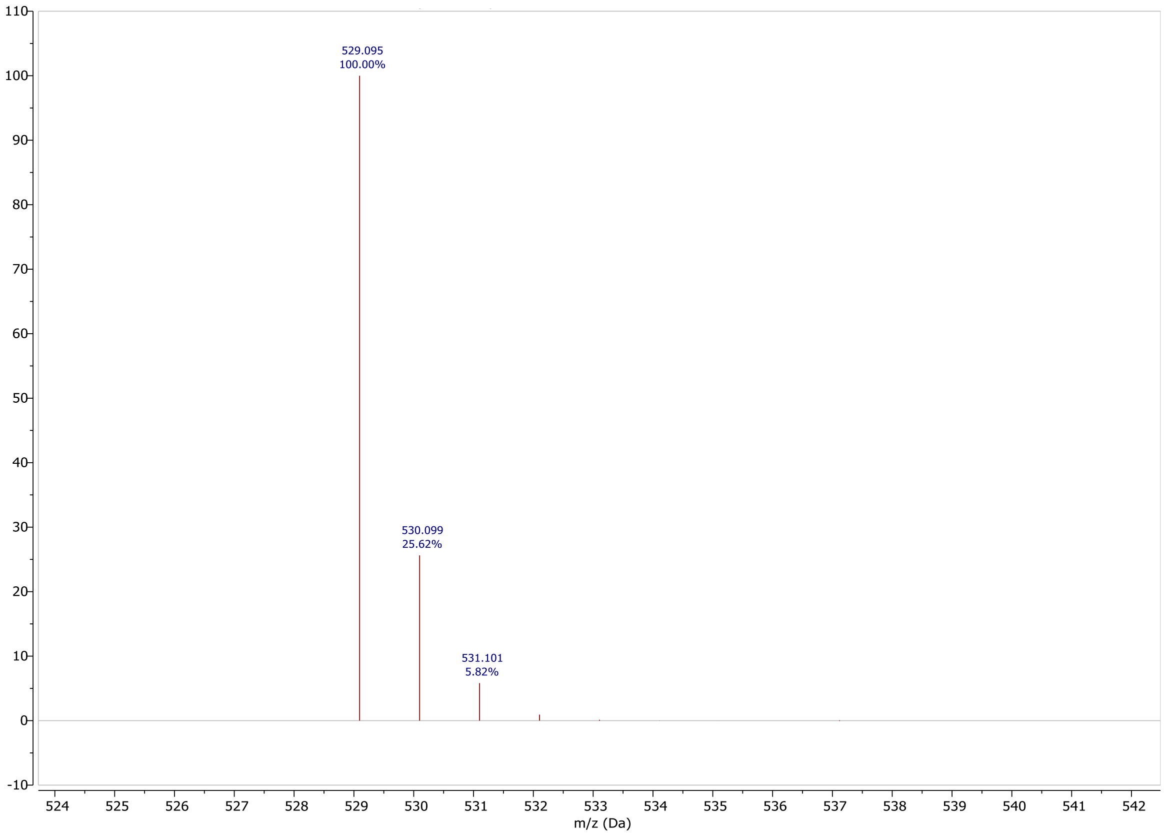

Figure S18: Mass spectrum of 3,3’,4’-Tri-O-methylellagic acid-4-O-β-Dglucopyranoside

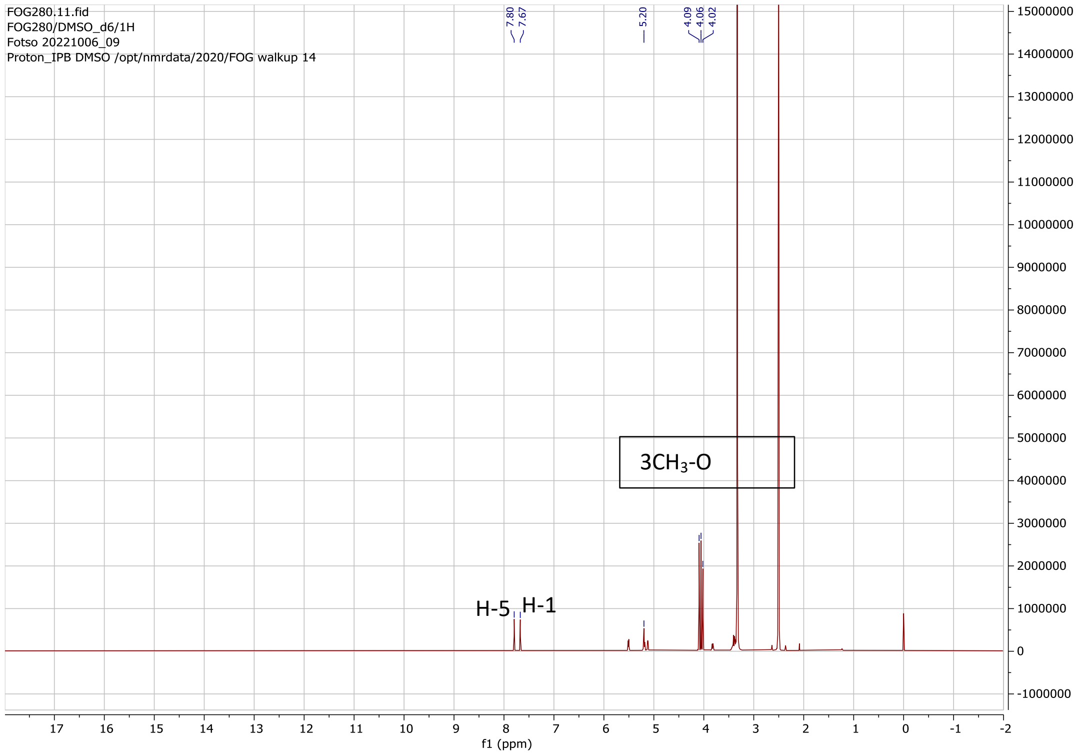

Figure S19: ^{1}H NMR spectrum of 3,3',4'-Tri-O-methylellagic acid-4-O-β-Dglucopyranoside

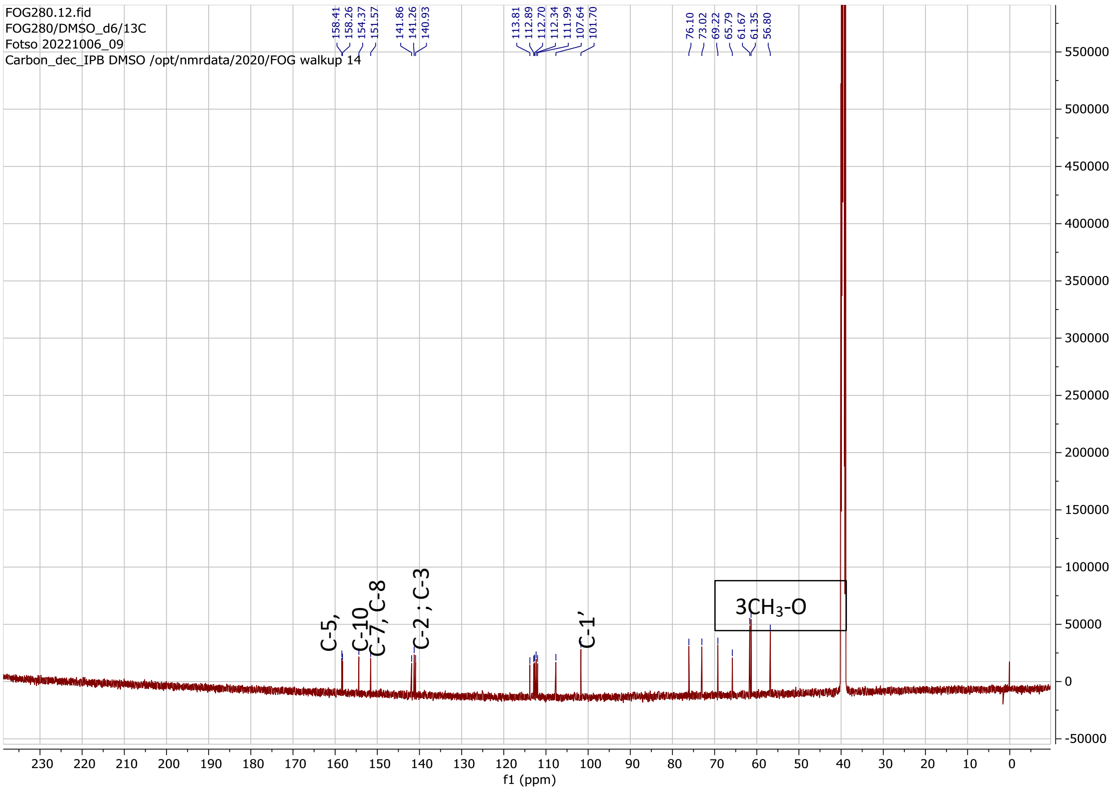

Figure S20: ^{13}C NMR spectrum of 3,3',4'-Tri-O-methylellagic acid-4-O-β-Dglucopyranoside

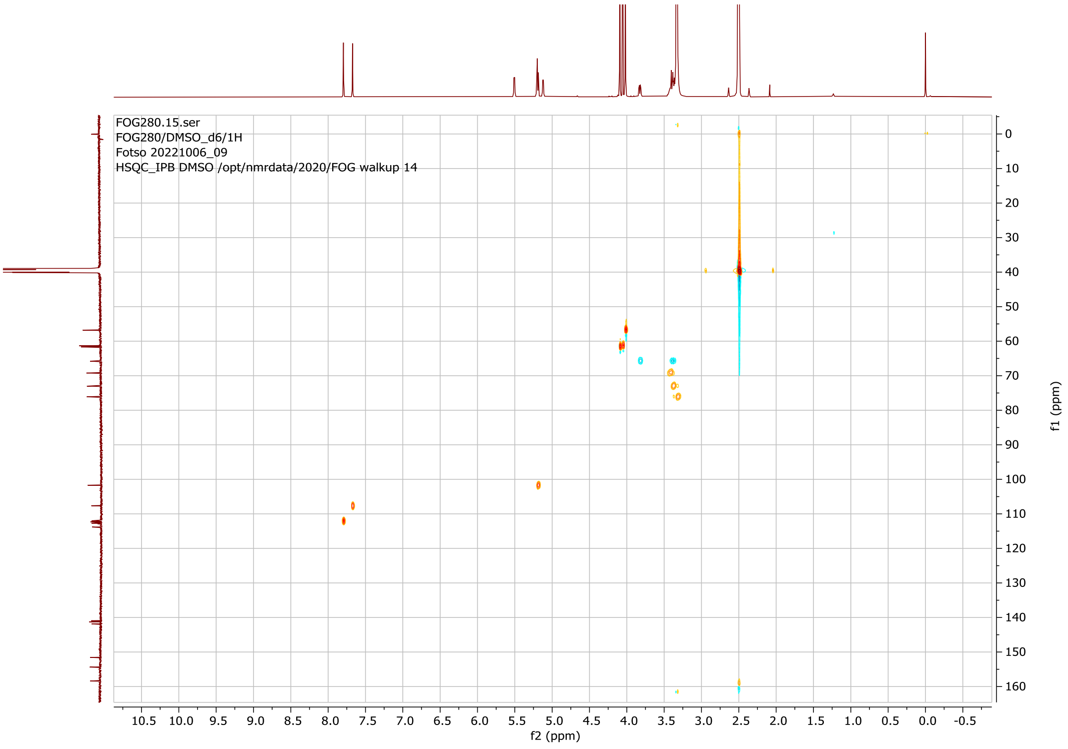

Figure S21: HSQC spectrum of 3,3’,4’-Tri-O-methylellagic acid-4-O-β-Dglucopyranoside

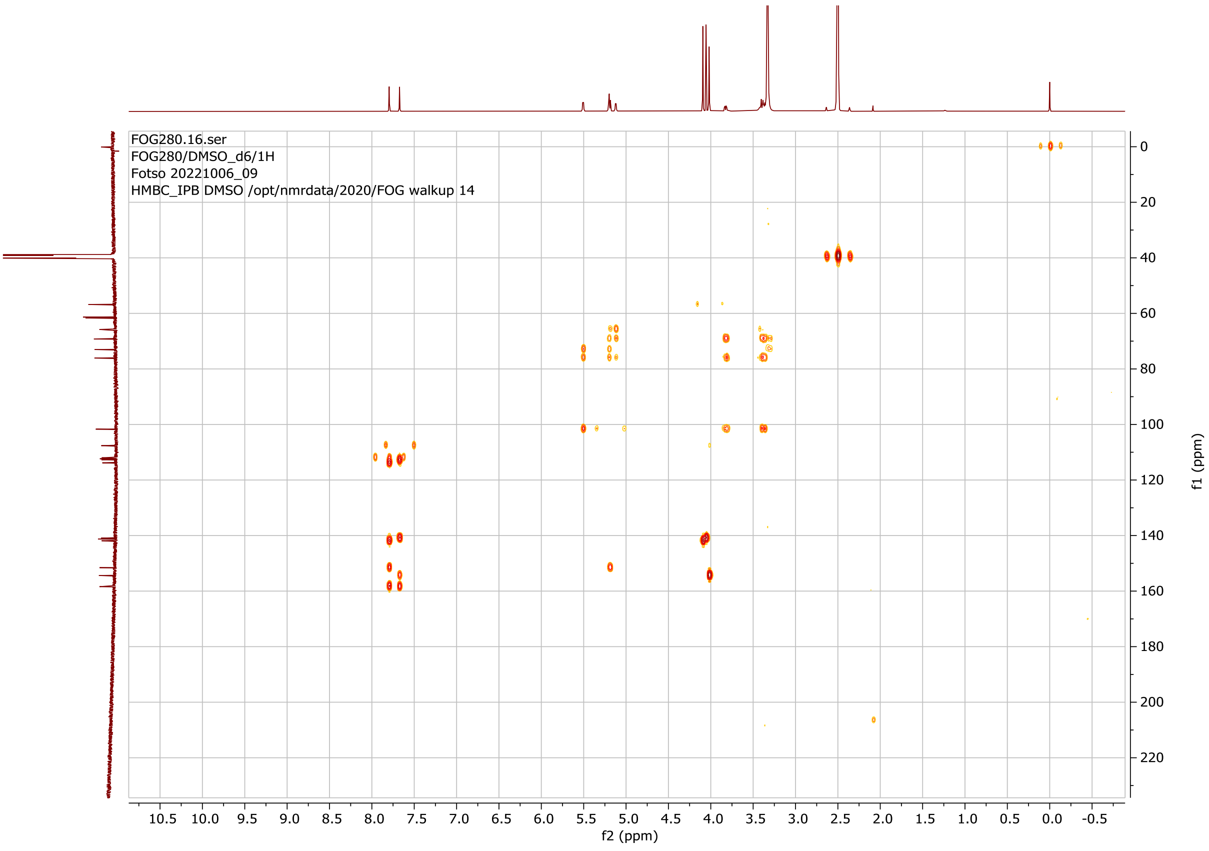

Figure S22: HMBC spectrum of 3,3’,4’-Tri-O-methylellagic acid-4-O-β-Dglucopyranoside

Table S1. ^{1}H-(400 MHz) and ^{13}C (100 MHz) NMR spectroscopic data of Compounds 1 2 and 3 recorded in $CDCl_3$.

	**Lupeol**		**Betulinic acid**		**Arjunolic acid**	
	**δc**	***δ*H**(m, *J* in Hz)	**δc**	***δ*H**(m, *J* in Hz)	**δc**	***δ*H**(m, *J* in Hz)
1	38.7		38.7		46,6	-
2	27.4		27.4		67,5	3,43(1H m)
3	79.0	3.12(1H, m)	79.0	3.21(1H, m)	75,6	3,13 (1H ; d ; J= 12,0 Hz)
4	38.9		38.8		42,6	-
5	55.3		55.3		48,6	-
6	18.5		18.3		18,9	-
7	34.3		34.3		33,3	-
8	40.8		40.7		40	-
9	50.4		50.5		47,1	-
10	37.2		37.2		37,4	
11	21.0		20.8		22,6	-
12	25.2		25.5		121,5	5,12 (1H ; t)
13	38.1		38.4		144,0	-
14	42.8		42.4		40,6	-
15	27.1		30.5		27,2	-
16	35.6		32.1		23,4	-
17	43.0		56.3		41,4	-
18	48.3		49.2		45,5	2,70 (1H ; dd ; J =11,3 ; 3,1 Hz)
19	48.0		46.9	3.02(1H,m)	46,0	-
20	150.9		150.4		30,4	-
21	29.9		29.7		32,1	-
22	40.0		37.1		31,9	-
23	28.0	0.94(3H ; *s)*	28.3	0.94(3H ; *s)*	63,9	3,26 (1H ; d ; J =10,1 Hz) 2,99(1H ; d ; J =10,1 Hz)
24	15.4	0.82(3H ; *s)*	22.1	0.82(3H ; *s)*	13,7	0,49(3H ; *s*)
25	16.1	0.84(3H ; *s)*	15.9	0.84(3H ; *s)*	16,9	0,87 (3H ; *s*)
26	16.0	0.94(3H ; *s)*	16.0	0.94(3H ; *s)*	16,8	0,66(3H ; *s)*
27	14.6	1.00(3H ; *s)*	14.8	1.00(3H ; *s)*	17,7	-
28	18.0	-	181.2	-	178,7	-
29	109.4	4.62(1H, d, 5) 4.50(1H, d, 5)	109.7	4.76(1H, d, 5) 4.63(1H, d, 5)	25,7	1,04 (3H ; *s*)
30	19.3	-	19.4	1.70	23,5	0,82 (3H ; *s*)

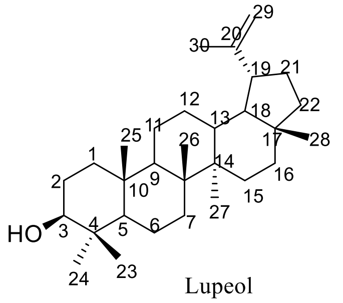
Lupeol

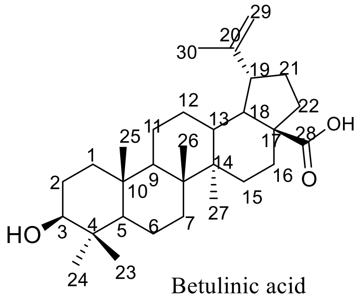
Betulinic acid

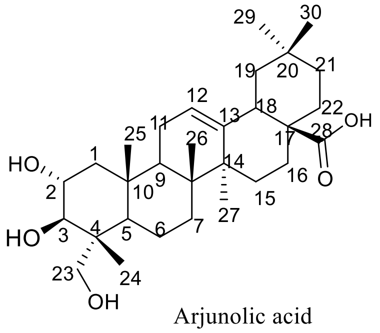
Arjunolic acid

Table S2. ^{1}H-(400 MHz) and ^{13}C (100 MHz) NMR spectroscopic data of Compounds 4 and 5 recorded in DMSO.

	3,3'-Di-O-methylellagic acid-4'-O-β-D-glucopyranoside		3,3',4'-Tri-O-methylellagic acid-4-O-β-Dglucopyranoside	
	δc	*δ*H (m, *J* in Hz)	δc	*δ*H(m, *J* in Hz)
1	112.7	-	112.8	-
2	111.9	7.50(1H, s)	111.9	7.80 (1H, s)
3	152.9	-	154.3	-
4	151.3	-	151.6	-
5	141.9	-	141.8	
6	111.1	-	107.6	-
7	158.5	-	158.2	
1'	111.9	-	112.3	-
2'	114.2	7.74 (1H, s)	113.8	7.67 (1H, s)-
3'	141.0	-	141.2	-
4'	141.6	-	140.9	-
5'	140.2	-	140.9	
6'	111.7	-	112.7	-
7'	158.4	-	158.4	-
1''	101.8	5.15 (1H, d, 8)	101.7	5.15 (1H, s)
-	61.7	4.09 (3H, s, 3-OMe)	61.6	4.09 (3H, s, 3-OMe)
-	61.1	4.05 (3H, s, 3'-OMe)	61.3	4.06 (3H, s, 3'-OMe)
-	-	-	56.8	4.02 (3H, s, 4'-OMe)

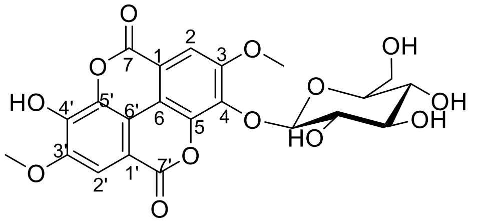

3,3'-Di-O-methylellagic acid-4'-O-beta-D-glucopyranoside

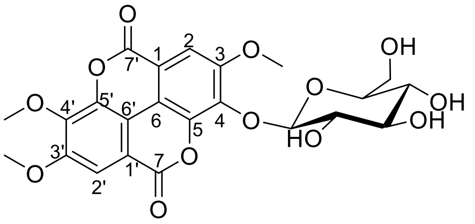

3,3',4'-Tri-O-methylellagic acid-4-O-beta-Dglucopyranoside
